# Pt nanoparticles under oxidizing conditions – implications of particle size, adsorption sites and oxygen coverage on stability†

**DOI:** 10.1039/d2na00490a

**Published:** 2022-09-13

**Authors:** Asfaw G. Yohannes, Karin Fink, Ivan Kondov

**Affiliations:** Institute of Nanotechnology, Karlsruhe Institute of Technology Hermann-von-Helmholtz-Platz 1 76344 Eggenstein-Leopoldshafen Germany; Steinbuch Centre for Computing, Karlsruhe Institute of Technology Hermann-von-Helmholtz-Platz 1 76344 Eggenstein-Leopoldshafen Germany ivan.kondov@kit.edu

## Abstract

Platinum nanoparticles are efficient catalysts for different reactions, such as oxidation of carbon and nitrogen monoxides. Adsorption and interaction of oxygen with the nanoparticle surface, taking place under reaction conditions, determine not only the catalytic efficiency but also the stability of the nanoparticles against oxidation. In this study, platinum nanoparticles in oxygen environment are investigated by systematic screening of initial nanoparticle–oxygen configurations and employing density functional theory and a thermodynamics-based approach. The structures formed at low oxygen coverages are described by adsorption of atomic oxygen on the nanoparticles whereas at high coverages oxide-like species are formed. The relative stability of adsorption configurations at different oxygen coverages, including the phase of fully oxidized nanoparticles, is investigated by constructing p–T phase diagrams for the studied systems.

## Abstract 2

Oxygen adsorption and oxide formation on platinum nanoparticles are investigated at different temperatures and pressures employing density functional theory and a thermodynamics-based approach.

## Graphical abstract

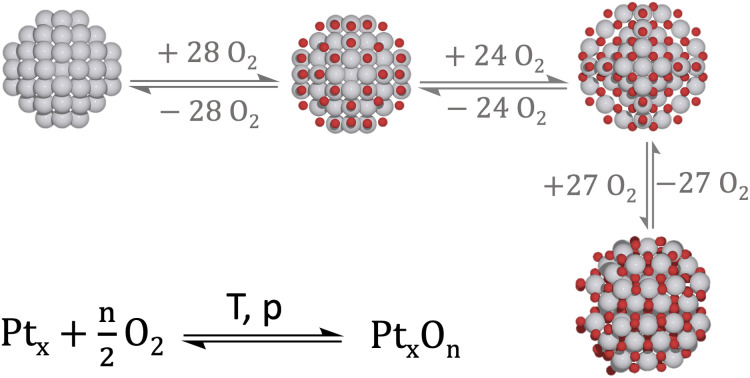

## 1 Introduction

Transition metals, such as platinum (Pt), are widely known for their catalytic performance in both oxidizing and reducing reactions and have been widely applied industrially for hydrocarbon oxidation,^1^ for abatement of automobile exhaust gases,^2,3^ in fuel cells^4^ and in catalytic reforming.^5^ The use of metallic nanoparticles as catalysts is motivated not only by their large active surface area but also by their special catalytic properties due to their particular size, shape and structure. For catalyst design, control over these latter variables implies the ability to manipulate the catalytic activity by introducing specific facets, edges and corners in the structure of the nanoparticles, which is essential for optimizing the activity and selectivity.^6^

Many industrially relevant catalytic reactions, relying on late transition metals as catalysts, often proceed in oxygen-rich operating conditions or involve oxygen as a reacting species on the surface of a metal catalyst. Acquiring a fundamental understanding of the nature of the interaction between metal surfaces and oxygen is key to explain the role of oxygen in a number of important catalytic chemical processes.^7^ Metal–oxygen interactions are manifested in the formation of different metal–oxygen surface states, such as surface-adsorbed atomic oxygen, surface oxide films, and even bulk metal oxides, for many metallic catalysts.^7^ Thereby atomic and molecular oxygen adsorption is regarded as oxygen activation for subsequent oxidation reactions on the catalyst surface and the reactivity of the catalyst is often represented as a function of the oxygen adsorbate coverage. A modeling study of small nanoparticles of different metals has shown that oxygen activation is enhanced substantially at active sites with low coordination numbers^8^ that raises the question about the stability of these sites against oxidation. Dissociation of molecular oxygen on the truncated octahedron platinum nanoparticles with 38, 79 and 116 atoms^9,10^ has been investigated using density functional theory (DFT) whereby it has been shown that barrierless oxygen dissociation occurs on the (111) facet accompanied by significant surface distortion. A preference for the edge bridge sites for atomic oxygen adsorption and an increase of the adsorption energy with atomic oxygen coverage have been found in a DFT study of cuboctahedral nanoparticles with 55, 147 and 309 atoms.^11^ Adsorption energy of atomic oxygen enters different scaling relations that, in combination with microkinetic modeling, enable prediction of the rates of various catalytic reactions^12^ involving this species.

When oxygen interacts with a metal surface, different oxide films can be formed depending on the partial pressure, temperature, and orientation of the metal surface. Numerous experimental and DFT studies have characterized oxide formation on different Pt surfaces,^13–18^ on the Pd(111) ^18–20^ and the Cu(111) surface.^21^ Pressure–temperature phase diagrams have been constructed for the extended Cu(111)–O ^21^ and Pt(111)–O ^15^ surface systems based on DFT data, as well as for extended Pd surfaces and Pd nanoparticles by using molecular dynamics.^22^ In all these studies surface oxide formation has been considered.^15,21,22^

CO oxidation on the surface of small Pt nanoparticles is known to accompany oscillatory behavior under realistic conditions and results in a change of the catalytic activity.^23,24^ This phenomenon would have consequences for the exhaust gas catalysis.^25,26^ Some of the hypotheses to explain this oscillatory behavior suggest cyclic oxidation-reduction^24,27^ and reversible surface phase transitions of Pt surfaces.^28^ There has been disagreement concerning whether the surface or subsurface Pt oxides are active.^18,29–32^ For example, Boubnov *et al.*^23^ have found that low-coordinated surface Pt sites on small Pt nanoparticles (nm) are oxidized at temperatures higher than 135 °C and become inactive for the CO oxidation.

A comprehensive *ab initio* study of adsorption of atomic oxygen will be therefore very useful to scrutinize these hypotheses by giving more insight and understanding of the oxygen–nanoparticle interactions. The produced data will enable the construction of phase diagrams of relevant Pt nanoparticles in oxygen environment to find the most stable oxygen adsorption configurations under experimental conditions and the limits of stability against formation of Pt oxide nanoparticles. To this end, we employ DFT to study atomic oxygen adsorption on four different Pt nanoparticles addressing all possible adsorption sites. To investigate the effect of oxygen coverage on the nanoparticles, after screening configurations with different occupied adsorption sites for different numbers of adsorbed oxygen atoms, we compute the oxygen adsorption energy. We determine how the adsorption energy depends on nanoparticle size, type and coordination number of the occupied adsorption sites and oxygen coverage. Using an approximation of the free energy based on the adsorption energies computed by DFT,^33,34^ we construct the p–T phase diagrams for all four nanoparticles. The phase diagrams allow us to examine the most stable adsorption configurations with varying the pressure and temperature and to find the conditions for which the metallic nanoparticles are not oxidized. In addition, structural reorganization of surface Pt atoms under increasing oxygen coverage will be detected and discussed.

## 2 Methods

The computations and data analyses in this work have been performed using FireWorks^35^ and the Atomic Simulation Environment (ASE).^36^

### 2.1 Structural models and DFT calculations

All DFT calculations have been performed using the Vienna *ab initio* simulation package (VASP) code^37,38^ with spin polarization. The core electrons have been described by using projector augmented wave (PAW) potentials.^39^ The wave functions (orbitals) of the valence electrons have been expanded using plane waves with an energy cutoff of 450 eV. The Perdew–Burke–Ernzerhof (PBE)^40^ generalized gradient approximation has been used to describe the exchange-correlation functional. The latter has been widely used for metallic systems^21,41,42^ including platinum nanoparticles. We expect that all adsorption energies will become somewhat higher when using the RPBE^43^ or PBE-vdW.^44^ However, the focus of this study is on the relative stability of the adsorbed species rather than on providing high-accuracy reference data or accuracy assessment of different charge density functionals.

The initial nanoparticle structures have been constructed from bulk fcc Pt with the experimental lattice constant of 3.92 Å corresponding to a nearest-neighbor distance of 2.77 Å. To be able to use the plane-wave VASP code for non-periodic Pt nanoparticles, the latter have been modelled in cubic cells with three-dimensional periodic boundary conditions. To ensure that the interactions between neighboring periodic images are negligible, a vacuum region along each of the three directions has been added so that the distance between two nearest surface atoms in neighboring images is at least 16 Å. The first Brillouin zone has been sampled by the gamma point only. Unconstrained geometry optimizations have been performed using the conjugate gradient algorithm until the maximum force on any atom was below 0.01 eV Å^−1^ and the electronic relaxation has been converged within 10^−6^ eV. The first-order Methfessel–Paxton smearing method^45^ with smearing width of 0.2 eV has been used.

For the adsorption energies discussed in Sections 3.1 and 3.2, the O_2_ energy has been calculated with the same DFT parameters outlined above. The O_2_ energy used in the free–energy diagrams in Section 3.3 has been corrected to compensate for the strongly overestimated O_2_ binding energy using a GGA density functional that has been reported *e.g.* in ref. 21 and in the references therein. First, the O_2_ dissociation energy has been calculated using the “hard” PAW pseudopotential with a kinetic energy cutoff of 900 eV and a Gaussian smearing parameter of 0.005 eV. Then, the zero-point energy (787.380 cm^−1^)^46^ has been subtracted from the experimental dissociation energy (493.687 kJ mol^−1^).^47^ Finally, the correction is found as the difference of the DFT-calculated O_2_ dissociation energy and the experimental O_2_ dissociation energy. The correction to the O_2_ energy obtained is 0.460 eV per atom that is in good agreement with the difference of 0.48 eV per atom reported in a similar DFT study.^21^

The Pt slabs, used in Section 3.1 to model the (111) and (100) extended surfaces on fcc Pt, have been constructed from bulk fcc Pt with a lattice constant of 3.92 Å by including three Pt layers in all space directions, *i.e.* using a 3 × 3 supercell. In the *z* direction, that is perpendicular to the slab surface, a vacuum layer of 12 Å has been included to both the top and the bottom slab surfaces. The adsorbed oxygen atoms have been added to the top layer in *z* direction. The positions of the atoms in the bottom layer in *z* direction have been kept fixed during relaxation performed using a Monkhorst–Pack k-point grid with (5, 5, 1) divisions. A similar slab model has been used elsewhere.^48–50^

Different PtO_2_ nanoparticles have been constructed from the most stable β-PtO_2_ bulk structure (orthorhombic, space group *Pnnm*)^51^ following this procedure: (1) repeat the atoms in the primitive cell along all cell vectors; (2) remove Pt and O atoms that are the most distant from the structure centroid and have the lowest coordination numbers so that the number of dangling bonds is minimized and the stoichiometry of PtO_2_ is maintained; (3) repeat step (2) until the target size is reached. By varying the parameters of the algorithm several different nanoparticles for each size have been obtained. The structures of these nanoparticles have been fully relaxed using Gaussian smearing of 0.05 eV until the difference between the energies of two subsequent ionic relaxation steps was less than 10^−4^ eV per Pt atom and the largest norm of the forces acting on the nuclei after the last ionic relaxation step was 0.05 eV Å^−1^. For each nanoparticle size the structure with the lowest total energy has been selected and its total energy has been used in the phase diagrams in Section 3.3. It is noted that we have not performed global optimization but a local relaxation of several different nanoparticles of the same size. The relaxed structures of the lowest-energy PtO_2_ nanoparticles are available in the ESI.†

The generalized coordination number^52,53^ has been used to describe the various individual adsorption sites on the Pt nanoparticle surface. The generalized coordination number 
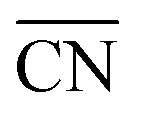
 of a surface site with *m* unique nearest neighbors is defined as:1
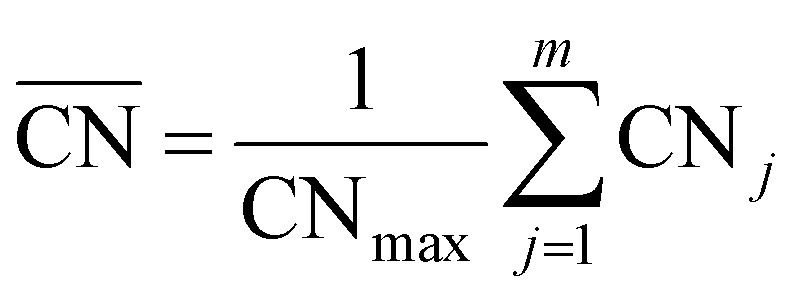
where CN_*j*_ is the coordination number of nearest neighbor *j* and CN_max_ is the number of nearest neighbors as if the site atoms would be in the bulk structure. The conventional coordination number of a surface site CN is defined as the total number of unique nearest neighbors of all atoms of the site. For example, for fcc metals, the CN_max_ is 12 for ontop sites, 18 for bridge sites, 22 for fcc and hcp sites and 26 for four-fold hollow (trough) sites. A Pt atom is considered a nearest neighbor of another Pt atom if the distance between the two atoms is less that 3.4 Å.

### 2.2 Calculating the adsorption energy and adsorption free energy

The adsorption energy of a single adsorbed oxygen atom on a Pt_*n*_ nanoparticle is defined as:2
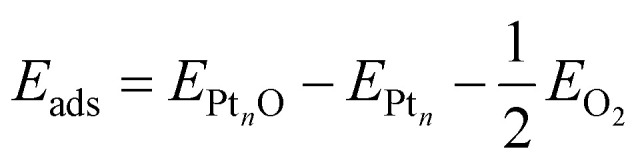
where *E*_O_2__ is the energy of an O_2_ molecule. The adsorption energy (per oxygen atom) for *N*_O_ oxygen atoms adsorbed on a nanoparticle *Ē*_ads_ is defined as3
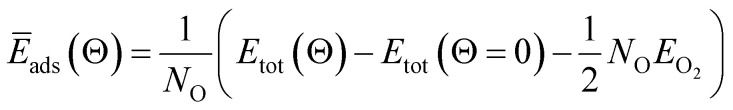
where *E*_tot_(*Θ*) and *E*_tot_(*Θ* = 0) are the total energies of the oxygen covered nanoparticle and the bare nanoparticle, respectively, and *N*_O_ is the number of adsorbed oxygen atoms. The oxygen coverage *Θ* of the nanoparticle surface is defined in monolayers (ML) so that 1 ML is realized when the number of adsorbed oxygen atoms is equal to the number of surface Pt atoms. According to eqn (2) and (3), a lower (or more negative) adsorption energy means a stronger binding of the adsorbates to the nanoparticle whereas a higher (or more positive) adsorption energy means a weaker binding.

When metal surfaces are exposed to oxygen, various structures can be formed depending on the partial pressure of oxygen and on the temperature, such as surface adsorbed oxygen, surface oxide films or bulk metal oxides.^21,54,55^*Ab initio* atomistic thermodynamics approach is applied to determine the effect of temperature (*T*) and pressure (*p*) on various surface-adsorbate structures under realistic experimental conditions.^21,54,55^ Using this approach, the most stable surface structure is determined at given temperature and oxygen partial pressure in the surrounding gas phase that enables the construction of a p–T phase diagram containing the stable regions of different phases.^21^ In this approach, the Gibbs free energy of adsorption Δ*G*_ads_(*Θ*, *T*, *p*) is defined in the equation:

4 Δ*G*_ads_(*Θ*, *T*, *p*) = *G*(*Θ*, *T*, *p*) − *G*(*Θ* = 0, *T*, *p*) − *N*_O_*μ*_O_(*T*, *p*)

where *G*(*Θ*, *T*, *p*) and *G*(*Θ* = 0, *T*, *p*) are the Gibbs free energies of the nanoparticle–oxygen complex at coverage *Θ* and of the bare nanoparticle, respectively, and *μ*_O_(*T*, *p*) is the chemical potential of gas-phase oxygen. The pressure dependence of the free energies *G*(*Θ*, *T*, *p*) and *G*(*Θ* = 0, *T*, *p*) can be neglected because the nanoparticle–oxygen complex and nanoparticle are much less compressible than the gas phase. In addition, the temperature dependence can be neglected by assuming that the oxygen overlayer and the bare nanoparticle are equally affected by the temperature and these temperature dependent terms will cancel each other in the calculation.^21,55^ Furthermore, the vibrational contributions to the free energies of the non-gas species usually nearly cancel in the total of the first two terms on the right-hand side of eqn (4).^21^ Therefore, the Gibbs free energies of the complex and of the bare nanoparticle can be approximated by the energies that can be readily computed using DFT. With these approximations, eqn (4) can be rewritten as

5 Δ*G*_ads_(*Θ*, *T*, *p*) ≈ *N*_O_(*Ē*_ads_(*Θ*) − *μ*_O_(*T*, *p*)

The chemical potential of gas-phase oxygen *μ*_O_(*T*, *p*) can be expressed as a function of temperature and pressure using the formula^21,54,55^6
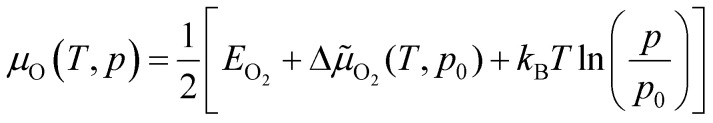
where *p*_0_ is the standard pressure (1 bar), *p* is the partial pressure of oxygen and *k*_B_ is the Boltzmann constant. The term Δμ̃_O_2__(*T*, *p*) includes the contributions from vibrations and rotations of O_2_, as well as the ideal gas entropy at standard pressure, and *E*_O_2__ is the electronic internal energy of the O_2_ molecule as calculated using DFT. The function Δμ̃_O_2__(*T*, *p*_0_) has been tabulated for temperatures in the range from 100 to 2000 K by using the Shomate equations with parameters based on experimental data^56^ (data provided in the ESI†).

## 3 Results

**Fig. 1:**
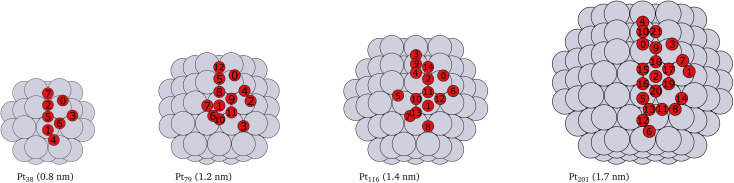
Cartoons of the structures of the studied nanoparticles. The size for every nanoparticle, shown in brackets, is defined as the longest inter-atomic distance. The platinum atoms are shown in gray color and the red circles indicate the locations of adsorbed oxygen atoms and the indices of the adsorption sites used in Tables 1, 2, 3 and 4. Multiply occurring sites are shown only once.

Platinum nanoparticles of sizes in the range of 1–10 nm have been employed as catalysts in experimental studies.^23,57,58^ Some studies have found that a particle size within the average size of 2 nm is optimal for high catalyst activity.^57–59^ On the other hand, truncated and cuboctahedron nanoparticles, that comprise both (111) and (100) facets in addition to edges and corners, show catalytic activity superior to regular octahedron and cubic nanoparticles.^41^ Several studies, for example,^7,9,53,60^ have been carried out on truncated octahedron nanoparticles. Therefore, we chose truncated octahedron nanoparticles Pt_38_, Pt_79_, Pt_116_ and Pt_201_ (shown in Fig. 1) with sizes in the 1–2 nm range.

### 3.1 Adsorption of single oxygen atoms

First, we consider single oxygen atoms adsorbed on the nanoparticles at all possible adsorption sites. This information allows us to determine the most preferred sites in the limit of low coverage and is needed for the screening in Section 3.2 to select lowest-energy configurations for adsorption at higher coverage.

The adsorption energies calculated using eqn (2) are summarized in Tables 1, 2, 3 and 4. Additionally, we have visualized the same data in Fig. 2. Because of the very complex behavior of the adsorption energy with variation of system size, facet/surface, site type and site location, we present the data also statistically in Fig. 2 and in Table 5. By computing the correlation coefficients (*r*^2^), we test how good different linear regression models for the adsorption energy as a function of site coordination numbers fit the available data. We choose a linear regression model assuming that the dependence of the adsorption energy on the coordination numbers is linear. This approach is well established for small adsorbate species, including atomic oxygen, on Pt nanoparticles.^52,53^ A small *r*^2^ means that the a linear model does not well fit the data whereas a large *r*^2^ implies (statistically) a good linear relationship.

**Table 1:** Adsorption energies (*E*_ads_) of single oxygen atoms on the Pt_38_ nanoparticle. The sites are described by their index #, see Fig. 1), type, location, coordination number (CN), generalized coordination number 
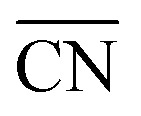
 and total number of sites (*N*_s_) of the given index

#	Site type	Site location	CN	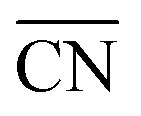	*E*_ads_ (eV)	*N*_s_
5	hcp	Corner	11	4.09	−1.69	24
2	Bridge	Edge 111–100	8	3.50	−1.61	24
6	fcc	Corner	10	3.68	−1.58	24
7	Trough	Corner	9	2.77	−1.27	6
3	Bridge	Edge 111–111	8	3.67	−1.23	12
0	Ontop	Corner	6	4.00	−1.03	24
1	Ontop	Terrace 111	9	6.00	−0.47	8
4	Bridge	Terrace 111	10	4.50	Unstable	48

**Table 2:** Adsorption energies (*E*_ads_) of single oxygen atoms on the Pt_79_ nanoparticle. The sites are described by their index #, see Fig. 1, type, location, coordination number (CN), generalized coordination number 
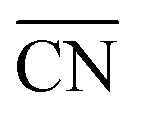
 and total number of sites (*N*_s_) of the given index

#	Site type	Site location	CN	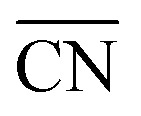	*E*_ads_ (eV)	*N*_s_
5	Bridge	Edge 111–100	8	3.61	−1.48	24
4	Bridge	Edge 111–111	9	4.33	−1.47	24
9	fcc	Corner	11	4.41	−1.40	48
10	fcc	Terrace	15	5.86	−1.40	8
8	hcp	Edge 111–100	11	4.45	−1.34	24
12	Trough	Corner	9	2.92	−1.32	6
11	hcp	Edge 111–111	14	5.68	−1.22	24
0	Ontop	Corner	6	4.08	−0.92	24
2	Ontop	Edge 111–111	7	5.00	−0.43	12
1	Ontop	Terrace 111	9	6.67	−0.12	24
3	Bridge	Terrace 111	10	4.94	Unstable	48
6	Bridge	Terrace 111	13	6.33	Unstable	24
7	Bridge	Terrace 111	11	5.39	Unstable	48

**Table 3:** Adsorption energies (*E*_ads_) of single oxygen atoms on the Pt_116_ nanoparticle. The sites are described by their index #, see Fig. 1, type, location, coordination number (CN), generalized coordination number 
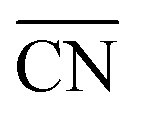
 and total number of sites (*N*_s_) of the given index

#	Site type	Site location	CN	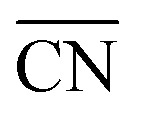	*E*_ads_ (eV)	*N*_s_
4	Bridge	Edge 111–100	9	4.33	−1.63	48
6	Bridge	Edge 111–111	8	3.89	−1.49	12
14	Trough	Corner	13	4.46	−1.40	24
12	fcc	Corner	10	4.14	−1.39	24
9	Bridge	Terrace 100	11	5.50	−1.33	72
11	hcp	Corner	12	4.95	−1.24	48
10	fcc	Edge 111–100	13	5.23	−1.23	24
13	hcp	Terrace	16	6.41	−1.01	8
0	Ontop	Corner	6	4.17	−0.87	24
2	Ontop	Edge 111–100	7	5.17	−0.56	24
3	Ontop	Terrace 100	8	6.33	−0.25	6
1	Ontop	Terrace 111	9	6.67	Unstable	24
5	Bridge	Terrace 111	10	5.00	Unstable	48
7	Bridge	Terrace 111	13	6.33	Unstable	24
8	Bridge	Terrace 111	11	5.50	Unstable	72

**Fig. 2:**
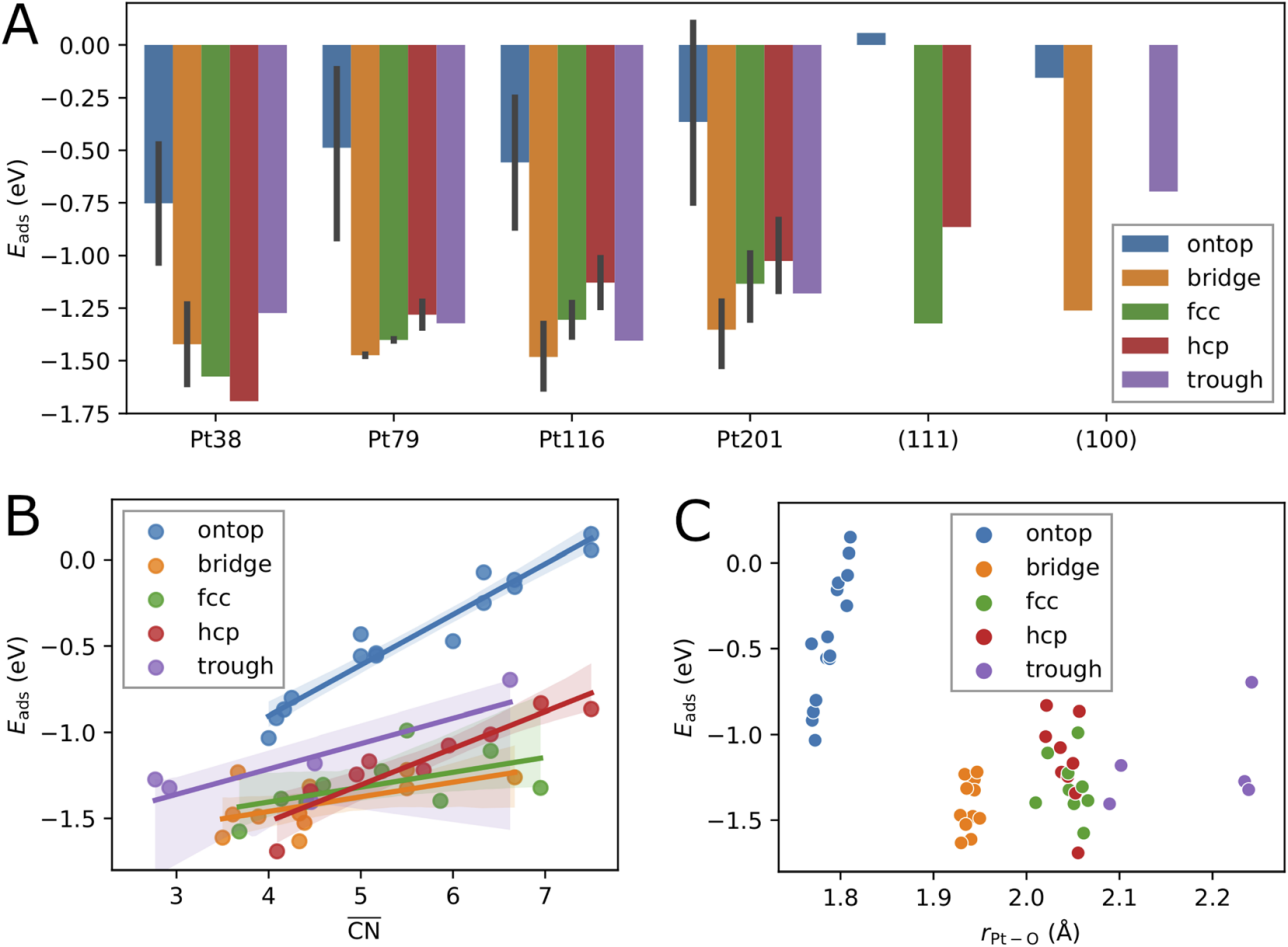
Correlation between the calculated adsorption energy *E*_ads_ and the system size (A), the generalized coordination number 
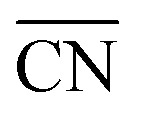
 (B), and the average Pt–O bond length *r*_Pt–O_ (C), for single oxygen atoms adsorbed at sites of different type. The black vertical bars denote minimum and maximum values.

### 3.1.1 Effects of the nanoparticle size and the adsorption site type

The variation of the adsorption energy with system size is shown in Fig. 2A. The ontop site adsorption energy is the highest compared to other sites that is also found for ontop adsorption on the extended (111) and (100) surfaces. On average, the adsorption energy increases with nanoparticle size and, with the exception of the Pt_116_ nanoparticle, approaches the adsorption energy at ontop sites on the extended surfaces. The correlation coefficient for a linear regression model is only 0.12 (see Table 5). Adsorption of O atoms at corner ontop sites is stable for all nanoparticle sizes and the adsorption energy is increasing monotonously. In contrast, ontop adsorption on the (111) terrace is stable with increasing energy on the smaller Pt_38_ and Pt_79_ nanoparticles while unstable on the large Pt_116_ and Pt_201_ nanoparticles. The latter is very similar to the ontop terrace adsorption on the extended (111) surface with the same CN = 9 and 
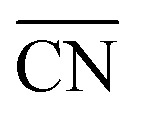
 = 7.5. Similarly to (111) terrace ontop adsorption, the adsorption energy at (100) terrace ontop site is increasing for Pt_116_ and Pt_201_ but is always lower than on the extended (100) surface.

For all nanoparticles, the adsorption at bridge sites on the (111) terrace is unstable with no exception. During a structural relaxation starting with an oxygen atom at such bridge sites, the oxygen atom migrates to a nearby three-fold hollow site (fcc or hcp). The same behavior is found for the bridge adsorption on the extended (111) surface. In contrast, bridge site adsorption at any locations other than the (111) terrace is the most stable on Pt_79_, Pt_116_ and Pt_201_. Bridge adsorption at edge sites is found particularly stable that agrees with the findings in previous DFT studies of oxygen on truncated octahedron nanoparticles.^7,9,10,60^ A similar site preference has been found for cuboctahedron nanoparticles.^11^ Only on the smallest Pt_38_, the adsorption at three-fold hollow sites is the most stable that is again in agreement with previous DFT studies.^7,9^ On average, the adsorption energy at bridge sites changes with nanoparticle size only slightly with no pronounced direction of change, that is described by the very low correlation coefficient of only 0.06. A similar behavior is found for the adsorption energy at four-fold hollow (trough) sites on the (100) facet. A linear regression analysis reveals a very slight increase of *E*_ads_ with nanoparticle size with a correlation coefficient of 0.22. In contrast, at fcc and hcp sites, *E*_ads_ strongly increases on average with nanoparticle size with correlation coefficients 0.72 and 0.60, respectively.

Adsorption energies on three-fold hollow sites (fcc and hcp) also exhibit large variations with the site locations. Adsorption on sites at corner locations is always more stable than at terrace and edge locations (see Tables 2, 3 and 4), while no regular preference between terrace and edge locations can be found. The energy differences are relatively small but, due to the large number of sites, the overall effect on the adsorption energy at high oxygen coverage can be very large. On the Pt_38_ nanoparticle, the hcp adsorption mode is the most stable followed by the fcc adsorption mode. The three-fold hollow site adsorption becomes less stable than the bridge site adsorption on Pt_79_, Pt_116_ and Pt_201_, and than the trough site adsorption on Pt_116_ and Pt_201_. On average, the adsorption energy at fcc and hcp sites strictly increases with nanoparticle size. The *E*_ads_ increase is weaker at fcc sites and stronger at hcp sites, as shown in Fig. 2A. This is in contrast to *E*_ads_ variation at bridge, ontop and trough sites where only a slight change with almost no trend can be found. Considering the comparable number of hollow sites and bridge sites at edge locations, we expect that the bridge and trough sites will more likely contribute to the most stable configurations at high oxygen coverage than the three-fold hollow sites.

**Table 4:** Adsorption energies (*E*_ads_) of single oxygen atoms on the Pt_201_ nanoparticle. The sites are described by their index #, see Fig. 1, type, location, coordination number (CN), generalized coordination number 
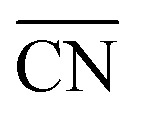
 and total number of sites (*N*_s_) of the given index

#	Site type	Site location	CN	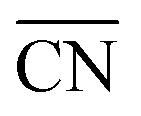	*E*_ads_ (eV)	*N*_s_
9	Bridge	Edge 111–100	9	4.39	−1.53	48
7	Bridge	Edge 111–111	9	4.44	−1.31	24
17	fcc	Corner	11	4.59	−1.31	48
10	Bridge	Terrace 100	11	5.50	−1.22	24
21	Trough	Corner	13	4.50	−1.18	24
18	hcp	Corner	12	5.09	−1.17	48
20	fcc	Terrace	15	6.41	−1.11	24
19	hcp	Edge 111–111	14	5.95	−1.08	24
15	fcc	Edge 111–100	13	5.50	−0.99	24
16	hcp	Terrace	16	6.95	−0.83	24
3	Ontop	Corner	6	4.25	−0.80	24
0	Ontop	Edge 111–111	7	5.00	−0.56	12
1	Ontop	Edge 111–100	7	5.17	−0.54	24
4	Ontop	Terrace 100	8	6.33	−0.07	6
5	Ontop	Terrace 111	9	7.50	0.15	8
2	Ontop	Terrace 111	9	6.92	Unstable	48
6	Bridge	Terrace 111	11	5.56	Unstable	48
8	Bridge	Terrace 111	11	5.67	Unstable	48
11	Bridge	Terrace 111	13	6.67	Unstable	48
12	Bridge	Terrace 111	13	6.67	Unstable	48
13	Bridge	Terrace 111	13	6.94	Unstable	48
14	Bridge	Terrace 111	10	5.17	Unstable	48

Adsorption at trough sites has a peculiarity that we briefly discuss here. In the smaller Pt_38_ and Pt_79_ nanoparticles, where the single trough site is located in the middle of the 100 facet, the distance from the adsorbed oxygen atom to all four Pt atoms of the site is the same, 2.235 Å and 2.239 Å, respectively. In the larger Pt_116_ and Pt_201_ nanoparticles, the four trough sites are located at the corners of the 100 facet. On these two nanoparticles the distances from the adsorbed oxygen atom to the site atoms are different, *e.g.* Pt_116_ the distances are 1.997 Å to the corner atom, 2.970 Å to the terrace atom, and 2.136 Å to the edge atoms. Very similar, on the Pt_201_ nanoparticle the distances are 2.017 Å to the corner atom, 2.917 Å to the terrace atom, and 2.144 Å to the edge atoms. Therefore, the average adsorption bond lengths on Pt_116_ and Pt_201_ for trough sites are by about 0.1 Å shorter than on the extended surface and on Pt_38_ and Pt_79_, as it is shown in Fig. 2C.

### 3.1.2 Effect of the local environment

While, on average, the adsorption energy of single oxygen atoms increases with nanoparticle size, the variation for the same site type with nanoparticle size is more noticeable. In the previous subsection, it has been shown that adsorption energies on sites at different locations can strongly vary. This is because the number of nearest neighbors of the site atoms (CN), as well as the average coordination number of the nearest neighbors of the site atoms, *i.e.* the generalized coordination number 
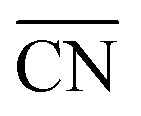
,^52^ depend on the specific location of the site of a given type. Therefore, in Fig. 2B, we consider here 
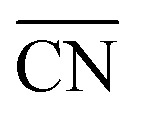
 as a structural descriptor and investigate its relation to the computed adsorption energy. The same relationship has been studied for oxygen and other adsorbate species on Pt_201_ nanoparticles and extended Pt surfaces.^52^

Plotting the generalized coordination number against the adsorption energies yields a very good linear relation suggesting a decrease of binding energy of the oxygen adsorbate to the adsorption site, *i.e.* increase of adsorption energy, with the generalized coordination number for all different site types. A particularly high correlation coefficient 0.94 is obtained for the ontop site adsorption. The same correlation coefficient has been found for OH adsorption on different Pt nanoparticles.^52^ Using the conventional coordination number CN results in significantly lower correlation coefficient of 0.80 that is similar to the finding in ref. 52. In Table 5, the correlation coefficients found for the linear models with nanoparticle size, CN and 
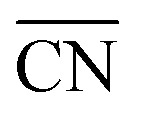
 as descriptors are summarized. While the correlation of *E*_ads_ with nanoparticle size is very weak, it is much stronger with CN, particularly when the data is grouped according to site types. When all data is included in the linear fit, the CN descriptor exhibits a poor correlation with correlation coefficient *r*^2^ = 0.06. A better correlation is obtained with 
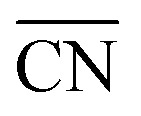
 for all sites except for the bridge sites where the correlation is of the same quality as with CN. When all data is included in the fit, the correlation coefficient is 0.39, *i.e.*
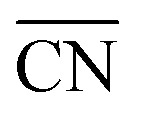
 is a more reliable descriptor for oxygen adsorption than CN. The thus defined linear regression model predicts lower adsorption energy for lower 
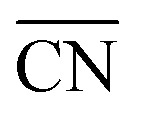
. However, the uncertainty in *E*_ads_ prediction by using 
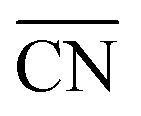
 alone is very large without using the site type as a second descriptor. As shown in Fig. 2B this uncertainty can be as large as 1 eV for 
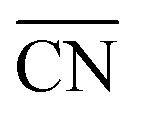
 in the range from 4 up to 7.5. In particular, adsorption energies at ontop sites are much higher than those at other site types with approximately the same 
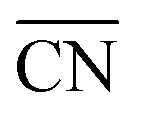
. An estimate of *E*_ads_ for adsorption on other nanoparticles can be more reliable if both 
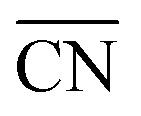
 and site type are determined. Fig. 2C shows that the adsorption energy does not correlate with the average Pt–O bond length as it has been found previously for O adsorption on Pt_201_.^60^ In contrast, the bond length correlates well with site type with very small variations owing to nanoparticle size effects and site locations. As shown in Fig. 2C, the average bond length can be used to determine ontop, bridge and hollow site types with high certainty.

**Table 5:** The square of the Pearson correlation coefficient between the adsorption energies *E*_ads_ and the coordination numbers (CN), the generalized coordination numbers 
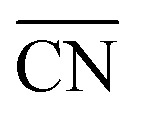
, and and the cluster size, for sites of different types. Adsorption on extended Pt surfaces is not considered in the relation with cluster size

Site type	Size	CN	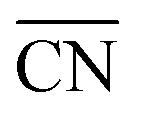
Ontop	0.12	0.80	0.94
Bridge	0.06	0.31	0.31
fcc	0.72	0.23	0.29
hcp	0.60	0.75	0.86
Trough	0.22	0.60	0.67
All types	0.08	0.06	0.39

### 3.2 Lowest-energy configurations with multiple oxygen adsorbates

**Fig. 3:**
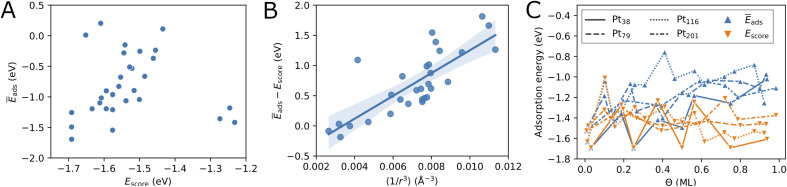
Relation between the score energy *E*_score_ and the adsorption energy *Ē* _ads_ (A) and between *Ē*_ads_ − *E*_score_ and 〈1/*r*^3^_*ij*_〉 (B) for the Pt_38_ nanoparticle. (C) Change of the lowest adsorption energy *Ē*_ads_ and the lowest energy score *E*_score_ with oxygen coverage. The lowest coverage shown corresponds to single-oxygen adsorption for every nanoparticle. The symbols denote the data points and the lines are only added as a guide to the eye.

In order to construct thermodynamic phase diagrams using eqn (5), it is important to find the most stable configurations with multiple adsorbed O atoms that are characterized by the lowest total adsorption energies per atom *Ē*_ads_. Due to the extremely large number of possible configurations this is a nontrivial task. As a first approximation, we consider configurations that include the maximum number of adsorbates of every site type. For the maximum number of sites of a given type, see Tables 1, 2, 3 and 4. Next, we neglect the contributions of adsorption at sites with very high single-atom adsorption energies. We neglect all ontop adsorption modes and unstable bridge-site adsorption modes and consider all other site types. For example, for Pt_38_ this approximation yields 31 different configurations. Applying the same approximation to Pt_201_ results in 1023 configurations. This is why there should be additional systematic screening of the configurations in order to reduce the number of candidate structures that would be feasible for DFT. Using the adsorption energies of single adsorbates from the previous section we construct an energy score:7
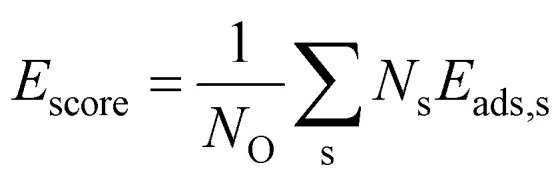
where s runs over all site types included in the particular configuration, *N*_s_ is the number of sites of type *s* and *E*_ads,s_ is the single oxygen adsorption energy at site s. To assess this screening criterion we have computed the *Ē*_ads_ for all 31 generated configurations on Pt_38_, as well as four additional configurations with partially occupied sites of a given type and location, and plotted *E*_score_ against *Ē*_ads_ for each configuration (see Fig. 3A). We find that some of the configurations with lowest *E*_score_ also have lowest *Ē*_ads_. Some configurations have much higher adsorption energies than suggested by the score and also three configurations with relatively high *E*_score_ have very low adsorption energies. This can be explained with collective electronic effects and lateral repulsion between closely adsorbed oxygen atoms. The latter effect should decay with powers of the inverse distance between oxygen atoms. Indeed, the difference between *Ē*_ads_ and *E*_score_ is in a good correlation with the mean of the third power of the inverse distance 〈1/*r*^3^〉, as seen in Fig. 3B. The quantity 〈1/*r*^3^〉 is defined as 〈1/*r*^3^〉 = ∑_*i*<*j*_ (1/*r*_*ij*_)/*N*_pairs_ with *N*_pairs_ the number of pairs of oxygen atoms (*i*, *j*). The good correlation suggests that the total adsorption energy is lower for larger distances between the adsorbed oxygen atoms. Thus, the quantity can be used as an additional criterion for selecting candidate configurations.

8 〈1/*r*^3^〉 [*E*_score_ − min (*E*_score_)]

Thus, after having performed calculations of all 35 configurations for Pt_38_, we calculated the adsorption energies of the first 50, 20 and 11 configurations for Pt_79_, Pt_116_ and Pt_201_, respectively, satisfying both criteria according to eqn (7) and (8). Additionally, for the larger nanoparticles, we added further adsorption configurations in that some site types are partially occupied and such that are not on the selection list but might be important considering their low adsorption energy of single O atoms. For example, the configuration on Pt_201_ with all 24 bridge sites (#7 in Fig. 1, CN = 9, 
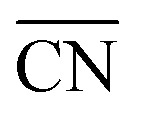
 = 4.44) occupied by O atoms is only at positions 24 and 27 in the sorted criteria according to eqn (7) and (8), respectively. However, this is the second most stable adsorption mode for single O atoms on Pt_201_ (see Table 4). After extending the list, we have computed the adsorption energy for 64, 35 and 36 configurations on Pt_79_, Pt_116_ and Pt_201_, respectively. A full list of the considered configurations is provided in the ESI.†

For every nanoparticle size, we have grouped the configurations with equal number of O atoms, *i.e.* according to the oxygen coverage in monolayers (ML). From every group we have selected the configuration with the lowest total adsorption energy. The relevant data is summarized in Table 6.

**Table 6:** Summary of the lowest-energy configurations with oxygen coverage *Θ* < 1 ML including the nanoparticle size (*N*_Pt_), number of adsorbed oxygen atoms (*N*_O_), coverage (*Θ*), the total number of considered configurations (*N*_c_), adsorption energy per O atom (*Ē*_ads_ in eV), the averaged Pt–Pt and Pt–O nearest-neighbor distances 〈*r*_Pt–Pt_〉_s_ (surface Pt atoms), 〈*r*_Pt–Pt_〉 (all Pt atoms) and 〈*r*_Pt–O_〉, respectively, in Å, the averaged Pt–Pt and Pt–O coordination numbers 〈CN_Pt–Pt_〉 and 〈CN_Pt–O_〉, respectively, and the site occupation number *N*_s_ and site index # according to Fig. 1. The ratings according to the two screening criteria are shown in the last two columns

*N*_Pt_	*N*_O_	Θ	*N*_c_	*Ē*_ads_	〈*r*_Pt–Pt_〉_s_	〈*r*_Pt–Pt_〉	〈*r*_Pt–O_〉	〈CN_Pt–Pt_〉	〈CN_Pt–O_〉	*N*_s_/#	*R*[eqn (7)]	*R*[eqn (8)]
38	1	0.03	7	−1.69	2.68	2.73	2.06	7.4	0.1	1/5	—	—
38	6	0.19	1	−1.36	2.67	2.75	2.25	7.6	0.6	6/7	—	—
38	8	0.25	2	−1.69	2.75	2.71	2.06	6.3	0.6	8/5	—	—
38	12	0.38	1	−1.42	2.74	2.75	1.95	7.6	0.6	12/3	—	—
38	16	0.50	2	−1.49	2.82	2.78	2.05	6.3	1.3	16/5	—	—
38	18	0.56	1	−1.18	2.84	2.82	2.02	7.6	1.3	6/7, 12/3	—	—
38	24	0.75	3	−1.26	2.89	2.95	1.99	7.6	1.9	24/5	—	—
38	30	0.94	3	−1.02	2.88	2.95	2.08	7.6	2.5	24/5, 6/7	—	—
79	1	0.02	10	−1.48	2.70	2.75	1.94	8.5	0.0	1/5	—	—
79	6	0.10	1	−1.18	2.70	2.75	2.25	8.5	0.3	6/12	37	1
79	8	0.13	2	−1.40	2.72	2.77	2.04	8.5	0.3	8/9	—	—
79	14	0.23	1	−1.07	2.71	2.80	2.12	8.5	0.6	8/10, 6/12	26	3
79	16	0.27	1	−1.33	2.76	2.77	2.03	7.9	0.6	16/9	—	—
79	24	0.40	9	−1.40	2.78	2.82	2.01	8.5	0.9	24/8	33	4
79	30	0.50	4	−1.15	2.77	2.82	2.10	8.5	1.2	24/8, 6/12	36	6
79	32	0.53	4	−1.17	2.80	2.81	1.97	8.5	0.9	24/4, 8/10	6	9
79	38	0.63	2	−1.03	2.86	2.84	1.99	8.5	1.2	24/4, 8/10, 6/12	8	7
79	48	0.80	7	−1.04	2.93	2.85	2.08	8.2	1.8	48/9	17	19
79	54	0.90	5	−1.11	2.96	2.91	2.06	8.5	2.1	48/9, 6/12	23	16
79	56	0.93	4	−0.97	2.88	2.86	2.01	8.5	1.8	24/4, 8/10, 24/8	16	15
116	1	0.01	11	−1.63	2.72	2.75	1.93	8.9	0.0	1/4	—	—
116	8	0.10	1	−1.04	2.73	2.79	2.01	8.9	0.2	8/13	38	2
116	12	0.15	1	−1.47	2.74	2.76	1.95	8.9	0.2	12/6	9	1
116	20	0.26	1	−1.07	2.75	2.77	2.00	8.9	0.4	12/6, 8/13	28	3
116	24	0.31	3	−1.08	2.75	2.78	2.09	8.9	0.6	24/12	18	4
116	32	0.41	1	−0.76	2.77	2.79	2.08	8.9	0.8	24/12, 8/13	32	9
116	36	0.46	2	−1.02	2.81	2.79	2.09	8.9	0.8	12/6, 24/14	13	11
116	44	0.56	1	−0.94	2.95	2.83	2.05	8.9	1.0	12/6, 24/14, 8/13	19	5
116	48	0.62	5	−1.07	2.79	2.80	1.95	8.9	0.8	48/4	1	52
116	56	0.72	2	−1.00	2.81	2.81	1.97	8.9	1.0	48/4, 8/13	4	37
116	60	0.77	3	−0.94	2.88	2.86	2.03	8.9	1.4	12/6, 48/11	31	21
116	68	0.87	1	−0.88	3.06	2.84	1.95	7.7	1.2	48/4, 12/6, 8/13	5	23
116	72	0.92	4	−1.25	2.96	2.88	1.97	8.9	1.4	48/4, 24/12	3	25
201	1	0.01	15	−1.53	2.72	2.77	1.93	9.4	0.0	1/9	—	—
201	24	0.20	3	−1.23	2.76	2.78	1.94	9.4	0.2	24/7	24	27
201	48	0.39	8	−1.28	2.81	2.81	2.00	9.4	0.6	24/17, 24/21	—	—
201	72	0.59	12	−1.04	2.90	2.83	2.04	8.7	1.1	48/17, 24/21	55	11
201	88	0.72	1	−1.08	2.91	2.85	2.02	9.4	1.2	48/17, 24/21, 8/20, 8/16	—	—
201	96	0.79	8	−1.20	2.94	2.87	1.98	9.4	1.2	48/9, 48/17	4	58
201	120	0.98	4	−1.11	2.99	2.87	2.00	9.1	1.6	48/9, 48/17, 24/20	11	48

Fig. 3C compares the adsorption energy *Ē*_ads_ with the energy score *E*_score_ for increasing oxygen coverage *Θ*. Overall, the adsorption energy *Ē*_ads_ increases with oxygen coverage. Particularly, the adsorption energy of all configurations with *Θ* > 0.5 ML is higher than −1.3 eV per O atom. For single-atom adsorption, the adsorption energies vary between around −1.7 and −1.5 eV while at maximum coverage these vary from about −1.3 and −0.8 eV. A very similar increase of the adsorption energy with the coverage has been found in a DFT study of oxygen adsorption on cuboctahedral Pt_55_, Pt_147_, and Pt_309_ nanoparticles^11^ although the changes found here exhibit much stronger oscillations. In the same work,^11^ a quantity that is equivalent to *E*_score_ has been found to behave very similar on average, apart from the oscillations with increasing the coverage that are found here for O adsorption on truncated octahedral nanoparticles. Furthermore, the increasing difference between *E*_score_ and *Ē*_ads_ is in agreement with that found in ref. 11 and suggests that *E*_score_ provides (statistically) good estimates of the adsorption energy for low and moderate oxygen coverage.

Furthermore, in Fig. 3C we compare the adsorption energy to the energy score *E*_score_ that does not include effects such as electronic band effects due to bonding multiple oxygen atoms and the interactions between adsorbate atoms. At the lowest coverage, *E*_score_ coincides with *Ē*_ads_. Then, in the low coverage range (*Θ* < 0.5 ML), *E*_score_ shows strong oscillations similar to *Ē*_ads_ and is relatively close to *Ē*_ads_. In the high coverage range (*Θ* > 0.5 ML), *E*_score_ is substantially lower than *Ē*_ads_. The higher adsorption energy can be explained with the effects of the large number of adsorbed atoms that are close to each other at high coverage. The energy score *E*_score_ does not include the latter effects and, therefore, does not change on average with the coverage, apart from the strong oscillations due to strongly different energies of the site types involved in the configurations. The increasing adsorption energy implies that configurations at high coverage will be stable only at sufficiently high oxygen chemical potential according to eqn (5).

After having introduced two screening criteria for the initially generated adsorbate configurations, one can naturally ask how successful the suggested selection criteria are by comparing the obtained lowest energy configurations. On average, in the cases of Pt_79_ and Pt_116_ the distance criterion is more successful for selecting low-coverage configurations, while the energy score criterion is more successful for selecting high-coverage configurations (see Table 6, last two columns). In the case Pt_201_ this also seems to be the case, though there are not many selected configurations in order to better support this finding. Overall, the distance criterion is more successful in selecting lowest-energy configurations on Pt_79_ than on Pt_116_. Five out of eleven lowest-energy configurations on Pt_79_ would have been missed if only the more simple energy score were used for screening. The Pt_79_ structures at 0.13 ML and 0.27 ML coverage and Pt_201_ structures at 0.39 ML and 0.72 ML coverage have been additionally considered due to their partial occupation, *i.e.* non-maximum possible *N*_s_.

### 3.3 Phase diagrams

As introduced above, the Pt nanocatalyst has applications in the context of the CO oxidation reaction, whereby the operation temperature can vary up to 800 K. An important question is whether the whole nanoparticles or their surfaces get oxidized to PtO_2_ at high temperature, particularly at 600 K.^18^ Furthermore, most of the studies of CO oxidation have been carried out either in ultra high vacuum (UHV) at pressure 10^−13^ bar or at pressures around the standard pressure (1 bar).^23,61^ Therefore, we vary both the pressure in a very large range enclosing the UHV pressures, as well as the standard pressure and consider different temperatures in the range from 100 K to 1800 K.

**Fig. 4:**
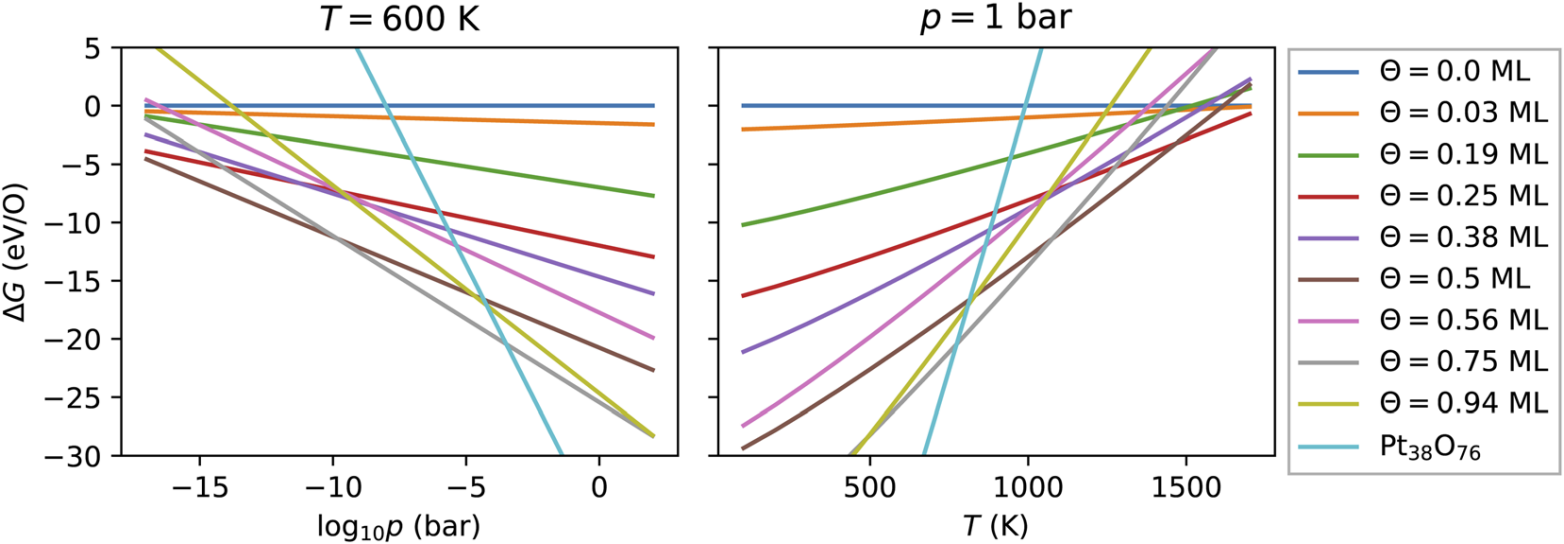
Adsorption free energy of all configurations with oxygen coverage below 1 ML on the Pt_38_ nanoparticle as a function of the pressure at *T* = 600 K and of the temperature at *p* = 1 bar. For comparison, the free energy of the fully oxidized nanoparticle Pt_38_O_76_ is shown.

For every nanoparticle and oxygen coverage, we select the configuration with the lowest energy. Then using eqn (5) we compute the free energy as a function of the temperature and the O_2_ partial pressure. In Fig. 4, we show the computed adsorption free energy at *Θ* < 1 ML for the Pt_38_ nanoparticle as a function of the pressure at *T* = 600 K and as a function of the temperature at standard pressure (1 bar). Additionally, we have shown the free energy of the fully oxidized Pt_38_O_76_ nanoparticle that was calculated using eqn (3) by replacing the total energy *E*_tot_(*Θ*) of the nanoparticle–adsorbate complex with the total energy of the Pt_38_O_76_ nanoparticle and setting *N*_O_ = 76.

Here, we explain the reason to consider PtO_2_ nanoparticles as reference instead of the bulk PtO_2_ phase. Comparing oxygen adsorption energies on extended surfaces to bulk PtO_2_ energies is possible due to the infinite reservoir of bulk Pt atoms below the surface. Beyond a critical oxygen chemical potential, an infinitely large number of bulk Pt atoms would be oxidized to form bulk PtO_2_ that would give rise to a vertical line in Fig. 4 (a detailed discussion can be found in ref. 62). In contrast, in the case of Pt nanoparticles, the reservoir of bulk Pt atoms in every nanoparticle is finite that is manifested by a non-vertical line in Fig. 4 for the fully oxidized phase. The number of surface atoms will increase proportionally with increasing the number of bulk atoms *via* the number of nanoparticles. Particularly, the Pt and PtO_2_ nanoparticles are less stable than the corresponding bulk phases due to their large surface energy. Though this effect is known, we illustrate it in the ESI† based on the available data and show that all nanoparticles become more stable with increasing their size. On the other hand, under experimental conditions, no extended platinum oxide bulk phase but partially and fully oxidized Pt nanoparticles have been observed.^23,27,58^ Consequently, we use the nanoparticle free energies and not those of the bulk phase in the thermodynamic stability analysis.

### 3.3.1 Adsorption at low and moderate oxygen coverage

Fig. 4 shows that from the eight lowest-energy configurations on Pt_38_ shown in Table 6 only three are absolutely stable at oxygen coverage of 0.25, 0.50 and 0.75 ML. These three configurations involve only the hcp sites with the occupation of 8, 16, and 24 O atoms, respectively. At *T* = 600 K, only at extremely low pressures *p* ≲ 10^−25^ bar, the adsorbate-free Pt_38_ nanoparticle is the most stable phase and at pressures *p* ≳ 10^−4^ bar the oxide nanoparticles become the most stable phase. Under UHV conditions, the phase with 0.5 ML coverage is found stable. Similarly, at standard pressure, for *T* ≲ 780 K and *T* ≳ 1760 K, the Pt_38_O_76_ nanoparticle and the bare Pt_38_ nanoparticle, respectively, are the most stable phases. We note that other processes might take place in this high temperature range that are not considered in this study: (i) decomposition of PtO_2_ to PtO and O_2_; (ii) evaporation of the PtO_2_ nanoparticles to PtO_2_ gas.^63–65^ However, temperatures beyond 800 K are too high compared to the typical operational temperature range of Pt nanocatalysts and we are aiming to explain and predict Pt nanoparticle stability against oxidation within the experimental temperature range relevant for catalytic applications. In addition, the region on the phase diagram including the PtO and gaseous PtO_2_ phases would be only in a narrow range of pressures.^64^

**Fig. 5:**
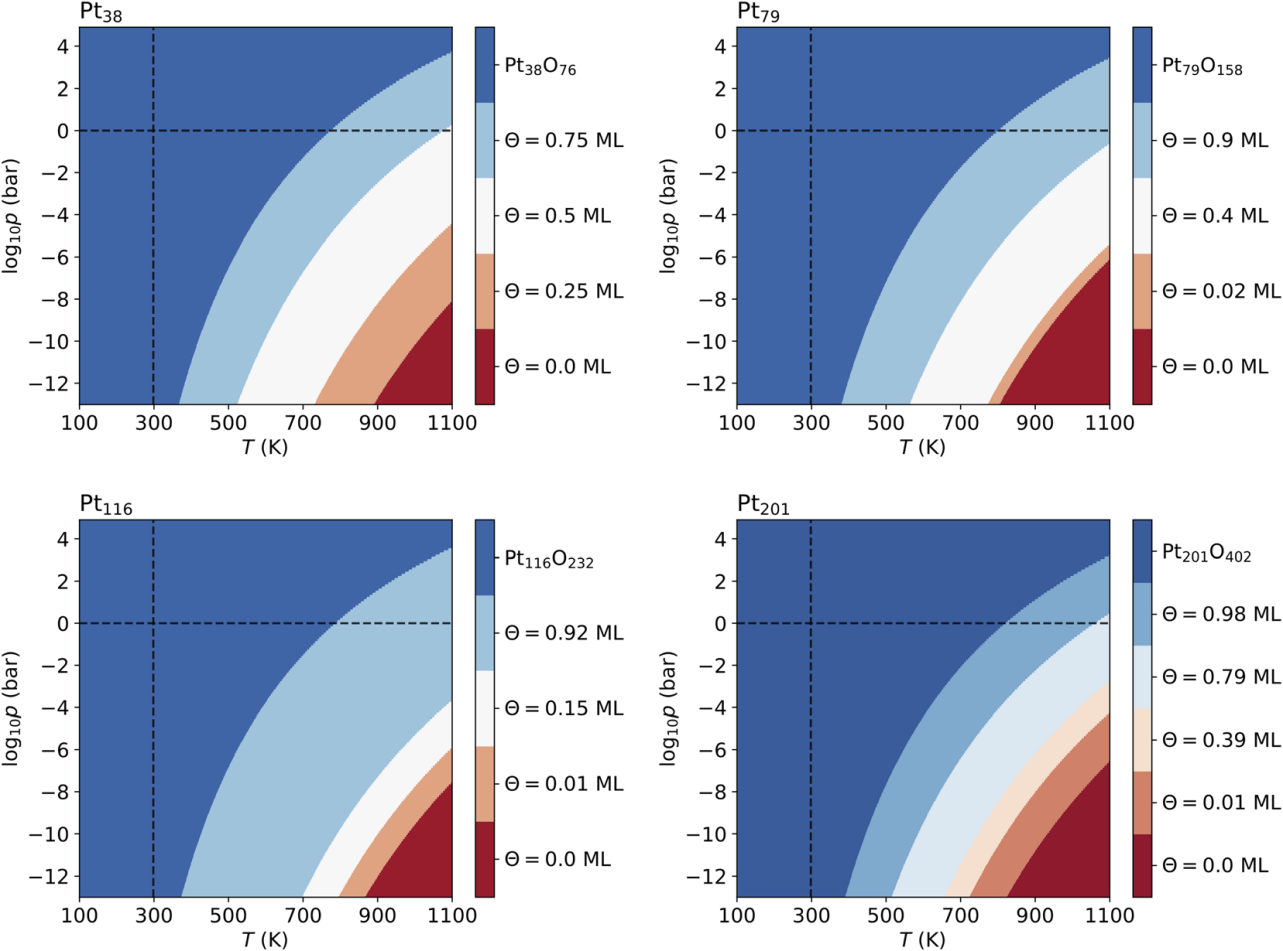
Phase diagrams displaying the most stable configurations with oxygen coverage up to 1 ML for the four nanoparticles. Standard temperature (*T* = 298.15 K) and standard pressure (*p* = 1 bar) are denoted by dashed lines. The pressure shown at the bottom of the diagrams corresponds to ultra high vacuum (UHV, *p* = 10^−13^ bar).

The computed p–T phase diagrams, shown in Fig. 5, include only the most stable phases. At standard pressure, the Pt_79_ nanoparticle is fully oxidized for temperatures above 800 K. The critical pressure for full oxidation of Pt_79_ at *T* = 600 K is *p* ∼ 10^−4^ bar. At standard pressure, the configuration at oxygen coverage of 0.9 ML is stable from ∼800 K up to ∼1150 K and under UHV conditions the same configuration is stable between 380 and 560 K. Between 560 and 770 K the configuration at coverage 0.4 ML is stable, followed by the configuration with one adsorbed oxygen atom at the (111)-(100) edge bridge site that is stable only up to 800 K after that the bare Pt_79_ nanoparticle becomes more stable.

On Pt_116_ the critical temperature of oxidation at standard pressure is ∼790 K which is very similar to the one found for Pt_38_ and Pt_79_. Again, the maximum pressure at 600 K for a stable metallic Pt_116_ oxygen-covered nanoparticle is ∼10^−4^ bar. In UHV the critical temperature is 380 K. The configurations with coverage of 0.92 ML is stable up to ∼700 K. The configuration with 0.15 ML coverage is stable between 700 and 800 K followed by the single-adsorbate complex up to 870 K. Adsorption at edge bridge sites dominates in all most stable configurations on Pt_116_, in contrast to Pt_38_ where only contributions of hcp adsorption modes, and to Pt_79_ where hcp, fcc and trough adsorption modes occur in the phase diagram.

On Pt_201_, the critical temperature of total oxidation at standard pressure is ∼820 K that is higher than any of the smaller nanoparticles. The maximal pressure for a non-oxidized Pt_201_ at 600 K is again ∼10^−4^ bar as has been found for all smaller nanoparticles. Four of the seven lowest-energy configurations occur in the phase diagram. Under UHV conditions, the configuration at coverage of 0.98 ML becomes stable for *T* ≳ 390 K. This configuration is stable for temperatures up to ∼512 K. At higher temperature, the configuration with coverage of 0.79 ML is formed and replaced only at ∼660 K by the configuration with *Θ* = 0.39 ML, followed by the complex with a single adsorbed oxygen atom (0.01 ML) occurring at 723 K that is stable up to 822 K. Notably, the two configurations at highest coverage of 0.79 and 0.98 ML combine (111)–(100) edge bridge and fcc adsorption modes while the configuration at moderate coverage of 0.39 ML combines corner fcc and trough adsorption modes.

### 3.3.2 Adsorption at high oxygen coverage

In the previous section, we have shown only data for coverage *Θ* ≤ 1 ML. Some of the selected configurations have oxygen coverage larger than 1 ML, *i.e.* the number of adsorbed oxygen atoms is larger than the number of surface atoms. The mean distance between the oxygen atoms is very small, however, some of these configurations are stabilized after a significant reorganization of the surface atoms and the adsorbed oxygen atoms during the structural relaxation. As we will show below, this reorganization does not only avoid the close initial distances between the adsorbed oxygen atoms but also leads to formation of an oxide layer on the surfaces of the nanoparticles.

In order to better quantify the degree of reorganization and oxidation of the nanoparticles with increasing the oxygen coverage, we investigated the lowest-energy structures, shown in Fig. S2 in the ESI, in more detail.† The full information about the reorganization towards nanoparticle oxidation is contained in the radial distribution function for the Pt atoms (Pt–Pt pair correlation function) and for the Pt and O atoms (Pt–O pair correlation function). In the following, we restrict the analysis to the averaged nearest-neighbor distance between Pt atoms 〈*r*_Pt–Pt_〉 and between Pt and O atoms 〈*r*_Pt–O_〉, that is the most relevant quantity describing this effect. Additionally, we will use the averaged Pt–Pt and Pt–O coordination numbers 〈CN_Pt–Pt_〉 and 〈CN_Pt–O_〉, respectively, that indicate the average number of Pt and O nearest neighbors, respectively, of a central Pt atom. The nearest-neighbor distance and coordination number correspond respectively to the position and the integral of the first peak of the radial distribution function. These quantities are shown in Tables 6 and 7 for all lowest-energy adsorption configurations. For the sake of readability, we will omit the word “averaged” in the following.

**Table 7:** Summary of the lowest energy configurations with oxygen coverage *Θ* > 1 ML including the nanoparticle size (*N*_Pt_), number of adsorbed oxygen atoms (*N*_O_), coverage (*Θ*), the total number of considered configurations (*N*_c_), adsorption energy per O atom (*Ē*_ads_ in eV), the Pt–Pt, and Pt–O nearest-neighbor distances 〈*r*_Pt–Pt_〉_s_ (surface Pt atoms), 〈*r*_Pt–Pt_〉 (all Pt atoms) and 〈*r*_Pt–O_〉, respectively, in Å, and the Pt–Pt and Pt–O coordination numbers 〈CN_Pt–Pt_〉 and 〈CN_Pt–O_〉, respectively. The site occupation number *N*_s_ and site index # according to Fig. 1 are shown in the last column

*N*_Pt_	*N*_O_	Θ	*N*_c_	*Ē*_ads_	〈*r*_Pt–Pt_〉_s_	〈*r*_Pt–Pt_〉	〈*r*_Pt–O_〉	〈CN_Pt_–_Pt_〉	CN_Pt_–_O_〉	*N*_s_/#
38	36	1.12	3	−1.06	2.92	2.93	2.00	7.6	2.5	24/5, 12/3
38	42	1.31	3	−1.04	2.99	3.00	2.04	7.6	3.2	24/5, 6/7, 12/3
38	48	1.50	3	−1.20	3.17	3.06	1.98	6.3	3.2	24/5, 24/6
38	54	1.69	3	−0.90	3.25	3.10	2.07	6.3	3.9	24/5, 24/6, 6/7
38	60	1.88	2	−0.68	3.28	2.98	2.02	5.1	3.8	24/5, 24/6, 12/3
38	66	2.06	3	−0.51	3.25	3.07	2.08	6.3	4.6	24/5, 24/6, 6/7, 12/3
79	62	1.03	2	−1.00	2.99	2.92	2.06	8.2	2.4	48/9, 8/10, 6/12
79	72	1.20	7	−1.22	3.12	2.92	1.99	6.7	2.4	24/5, 48/9
79	78	1.30	3	−0.96	3.11	2.98	2.00	7.9	2.4	24/5, 48/9, 6/12
79	80	1.33	5	−1.11	3.14	3.00	2.01	7.9	2.7	24/5, 48/9, 8/10
79	86	1.43	2	−0.66	3.02	2.99	2.09	6.7	3.3	24/4, 8/10, 24/8, 6/12, 24/11
79	96	1.60	3	−0.62	3.15	2.93	2.02	6.7	2.4	24/5, 48/9, 24/8
79	102	1.70	1	−0.49	3.04	2.88	2.07	6.1	3.4	24/5, 24/4, 24/8, 6/12, 24/11
79	104	1.73	2	−0.52	3.34	2.79	2.03	4.6	3.3	24/5, 24/4, 48/9, 8/10
79	128	2.13	1	−0.12	2.98	2.87	1.96	7.3	1.5	24/5, 24/4, 48/9, 8/10, 24/8
116	80	1.03	2	−1.13	2.97	2.88	1.99	8.9	1.7	48/4, 24/12, 8/13
116	84	1.08	2	−0.96	3.07	2.94	2.04	8.5	2.1	12/6, 24/14, 48/11
116	92	1.18	1	−0.88	3.09	2.98	2.04	8.9	2.3	12/6, 24/14, 48/11, 8/13
116	96	1.23	2	−0.96	3.03	2.92	2.01	8.9	2.1	48/4, 24/12, 24/10
116	104	1.33	1	−0.58	3.09	2.75	1.96	6.0	1.4	48/4, 48/11, 8/13
116	108	1.38	1	−0.73	3.28	2.79	1.99	5.4	2.3	48/4, 12/6, 48/11
116	116	1.49	1	−0.44	2.96	2.86	2.03	7.2	2.1	48/4, 12/6, 48/11, 8/13

First, we will consider the nearest-neighbor distances between surface Pt atoms 〈*r*_Pt–Pt_〉_s_ shown in Table 7. Their values vary from 2.92 Å to 3.34 Å for oxygen coverage from 1 up to 2 ML. For comparison, the nearest-neighbor distance in bulk fcc Pt, found in our DFT calculation, is 2.81 Å while in β-PtO_2_, the two nearest-neighbor Pt–Pt distances are 3.18 Å and 3.59 Å. The 〈*r*_Pt–Pt_〉 distance in the Pt_38_, Pt_79_, Pt_116_ and Pt_201_ nanoparticles has been found 2.68 Å, 2.70 Å, 2.72 Å and 2.71 Å, respectively.

**Fig. 6:**
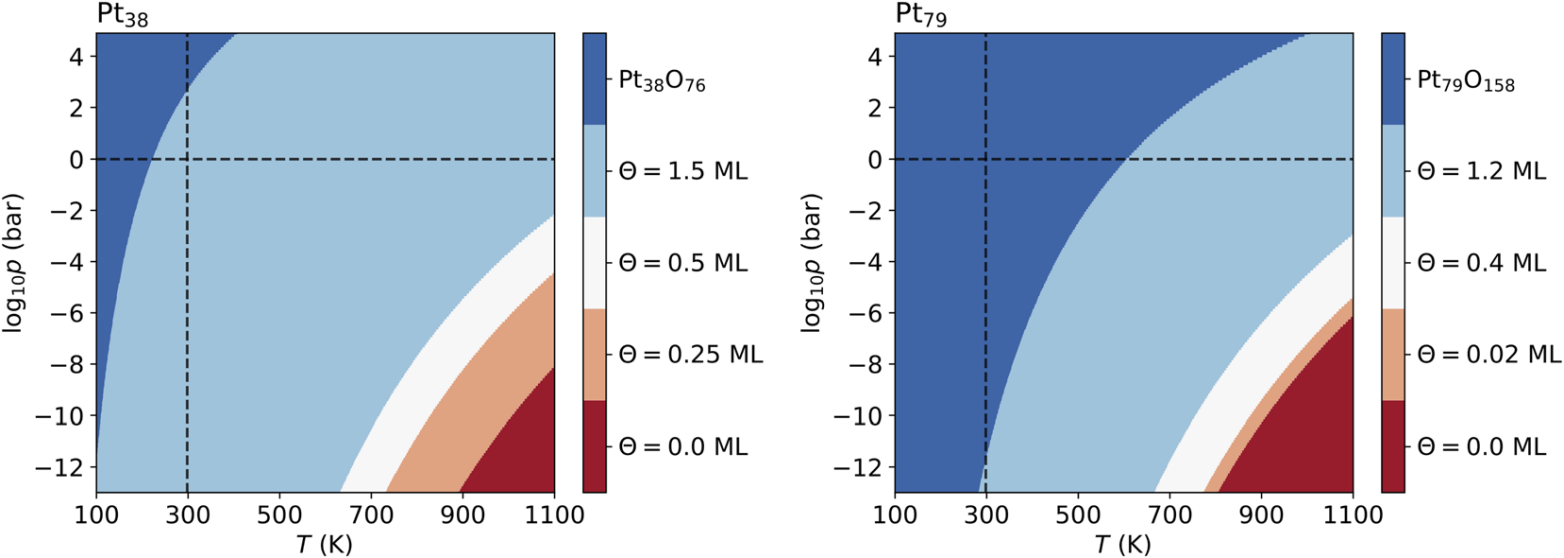
Phase diagrams showing the most stable configurations on Pt_38_ and Pt_79_ built with no restriction of oxygen coverage. Standard temperature (*T* = 298.15 K) and standard pressure (*p* = 1 bar) are denoted by dashed lines. The pressure shown at the bottom of the diagrams corresponds to ultra high vacuum (UHV, *p* = 10^−13^ bar).

For Pt_201_ no configurations with *Θ* > 1 ML have been selected using the two criteria according to eqn (7) and (8). The screening process for the Pt_116_ nanoparticles yields several configurations with *Θ* > 1 ML and seven of them are shown in Table 7 but none of these configurations contributes to the phase diagram. However, in the case of Pt_38_ and Pt_79_, we have found lowest-energy configurations with *Θ* > 1 ML occurring in the phase diagram shown in Fig. 6.

The lowest-energy configurations on Pt_38_ with oxygen coverage 1.12 and 1.31 do not show substantial reorganization of the nanoparticle indicating an oxidation that is evidenced by 〈*r*_Pt–Pt_〉_s_ below 3 Å. The configuration at *Θ* = 1.50 ML has the lowest absolute energy and appears prominently in the phase diagram (see Fig. 6A). This configuration has moderate degree of surface reorganization that is characterized by 〈*r*_Pt–Pt_〉_s_ Å. The configurations at coverage larger than 1.5 ML undergo a significant reorganization leading to oxidation of the corner platinum atoms. Characteristic for this reorganization are the high values of 〈*r*_Pt–Pt_〉_s_ that go beyond 3.25 Å.

The configuration at 1.03 ML on Pt_79_, with all fcc and trough sites occupied, exhibits a very weak reorganization of the surface Pt atoms, thus Pt_79_ preserves its characteristic shape. This is supported by the short nearest-neighbor distance between the surface Pt atoms (2.99 Å). The next lowest-energy configuration with 1.2 ML coverage, with 24 O atoms at (111)–(100) edge bridge sites and 48 O atoms at corner fcc sites is particularly stable and appears in the phase diagram (see Fig. 6B) although the initial average distance between the oxygen atoms is relatively short. The stabilization is due to a PtO_2_ oxide monolayer with a cage shape formed during structural relaxation involving all 36 Pt atoms from the twelve edges and the (100) facet (see Fig. S2 in the ESI†). The remaining 43 Pt atoms stay in a metallic Pt nanoparticle core enclosed in the oxide cage. For larger amounts of oxygen (>1.2 ML), no further stable phases can be found except for the fully oxidized Pt_79_O_158_ nanoparticle.

Overall, the degree of reorganization (oxidation) increases with oxygen coverage for all nanoparticles which is indicated by an increasing nearest-neighbor distance between the surface Pt atoms 〈*r*_Pt–Pt_〉_s_ in Tables 6 and 7 At coverage *Θ* < 0.5 ML, the nearest-neighbor distance is smaller than 3 Å in all considered configurations and nanoparticles. For *Θ* > 1 ML, the nearest-neighbor distance is larger than 3 Å in many configurations and larger than 2.8 Å in all configurations. The nearest-neighbor distance as a function of oxygen coverage is depicted in Fig. S3 in the ESI.†

## 4 Discussion

In the following we will compare the results to experimental X-ray photoelectron spectroscopy (XPS)^66^ and X-ray absorption spectroscopy (XAS) measurements^58,67,68^ on Pt nanoparticles.

### 4.1 Oxidation state

Svintsitskiy *et al.*^66^ have studied platinum oxide nanoparticles of average size of 2.3 nm (this is the size of Pt_201_O_402_ in this work) prepared by discharge sputtering of a Pt electrode in an oxygen atmosphere. Using XPS, they have found that the Pt(IV) oxide nanoparticles are stable at room temperature in UHV. After heating to 275 °C (548 K) the oxide nanoparticles completely convert to Pt(0) nanoparticles going through Pt(II) oxide. This is in overall agreement with the computed phase diagrams in Fig. 5 and 6 except that the oxygen-free phase (bare Pt nanoparticles) sets on at significantly higher temperature (800 K). The most likely reason for the overestimated transition temperature in our study is the PBE density functional used that is known to overestimate the adsorbate binding energy^69^ thus making the phases at low oxygen coverage more stable.

From XAS (EXAFS) spectra, Boubnov *et al.*^58^ have found O/Pt ratios between 0.32 and 0.90 for 1.2 nm and 2.1 nm Pt nanoparticles, depending on the oxidation state of the Pt nanoparticle. The highest O/Pt ratios of 0.66 and 0.90 after NO and CO oxidation reactions, respectively, have been found for the smallest 1.2 nm nanoparticle. The O/Pt ratio of 0.66 found after the NO oxidation reaction corresponds very closely (within 4%) to the phase with 0.90 ML coverage on the Pt_79_ nanoparticle that is most stable for coverage below 1 ML. The O/Pt ratio of 0.90 found after the CO oxidation reaction is very close (within 2%) to the one in the most stable phase at 1.20 ML coverage on the Pt_79_ nanoparticle. On the 2.1 nm nanoparticles, Boubnov *et al.*^58^ have found an O/Pt ratio of 0.32 after the CO oxidation reaction that does not correspond to any stable phase on the Pt_201_ nanoparticle (this corresponds to 0.52 ML oxygen on Pt_201_). It is noted that the 2.1 nm nanoparticle is considerably larger than Pt_201_ in this study that has a size of 1.7 nm.

### 4.2 Pt–Pt nearest-neighbor distances and coordination numbers

The Pt–Pt coordination numbers from EXAFS spectra^58^ found for the 1.2 nm nanoparticles are between 2 and 6 in the oxidized state and 7 and 9 in reduced metallic state, in agreement with our results. The Pt–Pt coordination number found here is 8.5 for the bare Pt_79_ nanoparticle and, with only few exceptions, for the phases for *Θ* < 1 ML (see Table 6). With increasing the oxygen coverage above 1 ML, the Pt–Pt coordination number decreases indicating smaller number of metallic bonds. For coverage larger than 1.20, the Pt–Pt coordination number is below 8 (see Table 7). For the most stable phase at 1.2 ML we find coordination number of 6.7. The coordination numbers between 2 and 6 found in ref. 58 must then correspond to more strongly oxidized nanoparticles than we have considered in this work. This is consistent with the Pt–Pt coordination number of 1.8 that we find in the fully relaxed PtO_2_ nanoparticle Pt_79_O_158_. This is somewhat smaller than the Pt–Pt coordination number (CN = 2) in the bulk β-PtO_2_ because in the bulk β-PtO_2_ every Pt atom has two nearest neighbors, both along the *x*-axis, whereas in the PtO_2_ nanoparticle some Pt atoms have only one nearest neighbor due the truncation. A similar decrease of the coordination numbers with the amount of adsorbed oxygen, starting with 8.9 for *Θ* = 0, is observed for the Pt_116_ nanoparticle.

The Pt–Pt nearest-neighbor distances from EXAFS spectra^58^ have been found between 2.67 and 2.74 Å for 1.2 nm Pt nanoparticles depending on the oxidation state of the Pt nanoparticle. These small differences between the Pt–Pt nearest-neighbor distances in the reduced and oxidized phases are comparable to the experimental standard deviation error of 0.02 Å.^58^ In contrast, the Pt–Pt nearest-neighbor distance found in the Pt_79_ nanoparticle increases with oxygen coverage from 2.75 Å to 3.00 Å. As discussed above, this is due to reorganization of the surface Pt atoms towards oxide formation that is supported by the found 〈*r*_Pt–Pt_〉_s_ =3.12 Å in the phase at *Θ* = 1.2 ML corresponding to the Pt–Pt nearest-neighbor distance within Pt–O–Pt bridges in β-PtO_2_.^67,68^ For comparison, we have found a slightly larger 〈*r*_Pt–Pt_〉_s_ = 3.14 Å in the fully relaxed Pt_79_O_158_ nanoparticle that is only slightly smaller from 3.18 Å in the bulk β-PtO_2_.

In a study of electrochemical Pt oxidation in solution, Imai *et al.*^67^ have reported a Pt–Pt nearest-neighbor distance of 2.75 Å and coordination number of 9.5 for a 1.9 nm Pt nanoparticles. This is in agreement with the Pt–Pt nearest-neighbor distance of 2.77 Å and Pt–Pt coordination number of 9.4 found here in the largest Pt_201_ nanoparticle. The authors of ref. 67 have attributed the increased Pt–Pt nearest-neighbor distance of 3.1 Å to the formation of PtO_2_ oxides: α-PtO_2_ for oxygen coverage up to 1.5 ML and the more stable β-PtO_2_ at higher oxygen coverage.

### 4.3 Pt–O nearest-neighbor distances and coordination numbers

The Pt–O nearest-neighbor distance found varies from 1.93 up to 2.25 Å. In the low-coverage configurations, the Pt–O nearest-neighbor distance is determined by the type of adsorption modes included in the configuration and the number of adsorbate species pertinent to each mode. The different adsorption modes have very characteristic distances between the surface Pt atoms and the adsorbate oxygen atoms, as discussed in Section 3.1 and shown in Fig. 2C. In the configurations at high oxygen coverage, surface oxidation and reorganization of the surface Pt atoms occur and lead to Pt–O nearest-neighbor distances closer to those in the Pt oxides. A strong variation of Pt–O nearest-neighbor distance from 2.01 up to 2.19 Å ^58^ and from 1.96 up to 1.99 Å ^68^ has been found from EXAFS spectra. Imai *et al.*^67^ have found a gradual transition from longer Pt–O bonds (2.2 Å) due to adsorbed oxygen species into shorter Pt–O bonds in Pt oxides (2.0 Å) based on XAFS measurements.

As expected, the Pt–O coordination number in the lowest-energy configurations is close to zero at low oxygen coverage (Table 6). The Pt–O coordination number increases with the oxygen coverage, as seen in Tables 6 and 7 In the Pt_38_ nanoparticle, for oxygen coverage larger than 0.94 ML the Pt–O coordination number is larger than 2.5. At *Θ* = 2.06 ML the Pt–O coordination number is 4.6 that is very close to 4.8 found in the Pt_38_O_76_ nanoparticle (CN = 6 in bulk PtO_2_) indicating a strong oxidation and formation of oxide-like compound. In the larger nanoparticles, the Pt–O coordination number also increases with O coverage initially but the increase is slower. The maximum Pt–O coordination numbers found in Pt_79_, Pt_116_ and Pt_201_ are 3.4, 2.3 and 1.6, respectively. These coordination numbers deviate significantly from those in the Pt_79_O_158_, Pt_116_O_232_ and Pt_201_O_402_ nanoparticles, that are 5.0, 5.1 and 5.2, correspondingly.

For 1.2 nm nanoparticles after NO oxidation reaction a Pt–O nearest-neighbor distance of 2.01 Å and a Pt–O coordination number of 2.7 have been found.^58^ These are close to 2.06 Å and 2.1, respectively, for the most stable configuration of Pt_79_ at 0.90 ML oxygen coverage that has a very close O/Pt ratio. Similarly, for the nanoparticles of the same size after CO oxidation the Pt–O nearest-neighbor distance and coordination number found are 2.11 Å and 2.5, while the values determined for the 1.2 ML stable configuration on Pt_79_ are 1.99 Å and 2.4, respectively. Because the computed 0.90 ML and 1.2 ML phases also occur in the phase diagrams of Pt_79_ (see Fig. 5 and 6), this agreement with the experimental EXAFS data is notable.

It is noted, that the effects of CO and NO used in the experiments^58^ are not considered in this work. They may have influence on the most stable adsorption phase both due to interaction and entropy contributions. In addition, the sizes of the nanoparticles 0.9, 1.9, 2.1 and 3.0 nm studied experimentally^58,67,68^ are different from those of the model nanoparticle studied here except for the 1.2 nm nanoparticles^58^ that correspond to Pt_79_. Moreover, under *in situ* experimental conditions the system may not be in thermodynamic equilibrium. Other factors not considered in our model are the silica, titania and alumina supports^58,68^ and the metallic platinum support and solvent environment^67^ that may also influence the measured nanoparticle properties.

## 5 Conclusions

In this work, we have studied truncated octahedron platinum nanoparticles Pt_38_, Pt_79_, Pt_116_ and Pt_201_ exposed to oxygen using density functional theory. We have computed the adsorption energy for different amounts of adsorbed atomic oxygen on the nanoparticles considering all possible adsorption modes. In the case of single-atom adsorption, we have found that ontop adsorption is either unstable or has the highest adsorption energies compared to any other adsorption modes. Bridge adsorption at the (111) terrace sites is unstable without exceptions. The top-three most preferred adsorption sites are located at the edges and at the corners. Overall, we have found only weak correlation between adsorption energy and nanoparticle size as well as between the adsorption energy and coordination numbers (either generalized or the conventional). The latter correlation becomes more pronounced when the adsorption modes are considered separately. We find that the generalized coordination number provides a better description (based on a linear fit) for the adsorption energy than the conventional coordination number if every adsorption site type is taken separately.

Considering all stable single-atom adsorptions and neglecting ontop adsorptions, we have generated all possible adsorption configurations at maximum occupancy and used two different screening criteria to further reduce the number of configurations. The distance criterion is more successful for selecting configurations at low coverage, whereas the energy criterion works better for configurations at high coverage. Due to the variance of the criteria, there may be still some statistical uncertainty that can be further reduced by increasing the number of output structures. Molecular dynamics or Monte Carlo sampling methods can be considered to replace the screening, especially for larger nanoparticles where the generation of the input structures for screening may become infeasible due to their extremely huge number. At each oxygen coverage between 0 and 2 ML, we have selected the lowest-energy configuration and constructed thermodynamic p–T phase diagrams for the four systems.

Overall, with increasing the amount of oxygen, we have observed stronger reorganization of the surface layer evidenced by the increasing nearest-neighbor distance between the surface Pt atoms. This reorganization leads to the formation of oxide-like surface compounds on Pt_38_ and Pt_79_ for *Θ* > 1 ML that are characterized by Pt–Pt and Pt–O nearest-neighbor distances and coordination numbers becoming closer to those in the corresponding PtO_2_ nanoparticles. Overall, the computed phase diagrams and the structural data is in good qualitative agreement with XPS and XAS experimental measurements. For example, the nanoparticles are fully oxidized at room temperature both at standard pressure and highly oxidized in ultrahigh vacuum, that is in agreement with the experimental findings. The computed structural data for Pt_79_, for which direct comparison has been possible, are in very good quantitative agreement with XAS structural characterization data. In particular, the degree of oxidation and structural data for the two most stable phases on Pt_79_ correspond to these found in experiments with 1.2 nm nanoparticles.

This study has focused on the stability of the nanoparticles–oxygen system with regard to thermochemical conditions. In future, the presented approach can be readily extended to study stability of the system in various electrochemical conditions, *i.e.* for the construction of Pourbaix diagrams.

## Author contributions

Conceptualization, K. F.; data curation, A. G. Y. and I. K.; formal analysis, A. G. Y. and I. K.; funding acquisition, I. K. and K. F.; investigation, A. G. Y. and I. K.; methodology, A. G. Y., I. K. and K. F.; project administration, I. K. and K. F.; resources, I. K. and K. F.; software, A. G. Y. and I. K.; supervision, I. K. and K. F.; validation, I. K. and K. F.; visualization, A. G. Y. and I. K.; writing – original draft, A. G. Y. and I. K.; writing – review & editing, A. G. Y., I. K. and K. F.

## Conflicts of interest

There are no conflicts to declare.

## Supplementary Material

NA-004-D2NA00490A-s001
