# Supplementary material for: Pt nanoparticles under oxidizing conditions – implications of particle size, adsorption sites and oxygen coverage on stability
Source: Nanoscale Adv. 2022 Sep 13;4(21):4554–69. doi: 10.1039/d2na00490a (PMC9595194; doi:10.1039/d2na00490a)
Supplement: NA-004-D2NA00490A-s001 [file NA-004-D2NA00490A-s001.pdf]

Electronic Supplementary Material (ESI) for Nanoscale Advances.
This journal is © The Royal Society of Chemistry 2022

Pt nanoparticles under oxidizing conditions – implications of particle size, adsorption sites and oxygen coverage on stability (Supporting information)

Asfaw G. Yohannes,[†,‡,¶] Karin Fink,[†] and Ivan Kondov*,[‡]

†*Institute of Nanotechnology, Karlsruhe Institute of Technology (KIT), Hermann-von-Helmholtz-Platz 1, 76344 Eggenstein-Leopoldshafen, Germany*

‡*Steinbuch Centre for Computing, Karlsruhe Institute of Technology, Hermann-von-Helmholtz-Platz 1, 76344 Eggenstein-Leopoldshafen, Germany*

¶*Present address: Department of Chemistry, University of Calgary, 2500 University Drive NW, Calgary, Alberta, T2N 1N4, Canada*

E-mail: ivan.kondov@kit.edu

Contents

List of Figures

S1 Cartoons of the PtO_2 nanoparticles. Platinum and oxygen atoms are shown in gray and red colors, respectively 4

S2 Selected lowest-energy configurations at high oxygen coverage exhibiting higher degree of structural reorganization manifested by averaged Pt-Pt nearest-neighbor distance $\langle r_{\mathrm{Pt-Pt}} \rangle_{\mathrm{s}} > 3$ Å (see Table 7). 5

S3 Dependence of the averaged nearest-neighbor distance between the surface platinum atoms $\langle r_{\mathrm{Pt-Pt}} \rangle_{\mathrm{s}}$ on the oxygen coverage Θ. 6

List of Tables

S1 Structural properties of the computed configurations (see Table S2) for configuration descriptions: Coverage Θ (in monolayer, ML), adsorption energy per O atom $\overline{E}_{\mathrm{ads}}$ (in eV), averaged Pt-Pt nearest-neighbor distance for surface Pt atoms $\langle r_{\mathrm{Pt-Pt}} \rangle_{\mathrm{s}}$ (in Å), averaged Pt-Pt nearest-neighbor distance for all Pt atoms $\langle r_{\mathrm{Pt-Pt}} \rangle$ (in Å), averaged Pt-O distance $\langle r_{\mathrm{Pt-O}} \rangle$ (in Å), averaged Pt-Pt coordination number $\langle \mathrm{CN}_{\mathrm{Pt-Pt}} \rangle$, averaged Pt-O coordination number $\langle \mathrm{CN}_{\mathrm{Pt-O}} \rangle$. 6

S2 Full list of the computed configurations specified by the number of oxygen atoms N_{s} of the specified site, coordination number CN and generalized coordination number $\overline{\mathrm{CN}}$. The list ordering is the same as in Table S1. 16

S3 Averaged Pt-Pt nearest-neighbor distances $\langle r_{\mathrm{Pt-Pt}} \rangle$ (in Å), averaged Pt-Pt coordination numbers $\langle \mathrm{CN}_{\mathrm{Pt-Pt}} \rangle$, averaged Pt-O nearest-neighbor distances $\langle r_{\mathrm{Pt-O}} \rangle$ and averaged Pt-O coordination numbers $\langle \mathrm{CN}_{\mathrm{Pt-O}} \rangle$ calculated for different model systems. 29

S4 Formation energies of the Pt nanoparticles and two extended Pt surfaces relative to bulk fcc Pt, and the formation energies PtO_2 nanoparticles relative to the formation energy of bulk PtO_2, each in eV per Pt atom. 29

Listings

S1 Structure of the bulk β-PtO_2 (in CIF format) used to create the PtO_2 nanoparticles 30

Tabulating $\Delta\widetilde{\mu}_{\mathbf{O_2}}(T, p_0)$

The data set and the formulas used to tabulate the O_2 chemical potential at standard pressure for different temperatures $\Delta\widetilde{\mu}_{O_2}(T, p_0)$ that is used in Eq. (6) can be found under this link: `https://webbook.nist.gov/cgi/cbook.cgi?ID=C7782447` in the section **Gas phase thermochemistry data**.

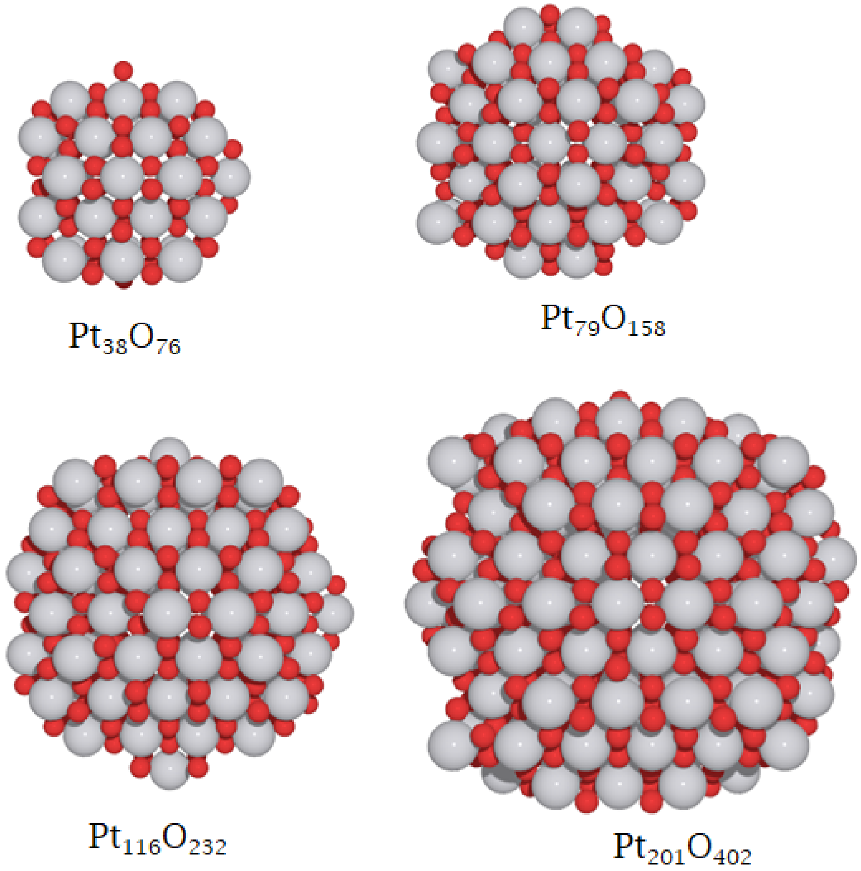

Figure S1: Cartoons of the PtO_2 nanoparticles. Platinum and oxygen atoms are shown in gray and red colors, respectively

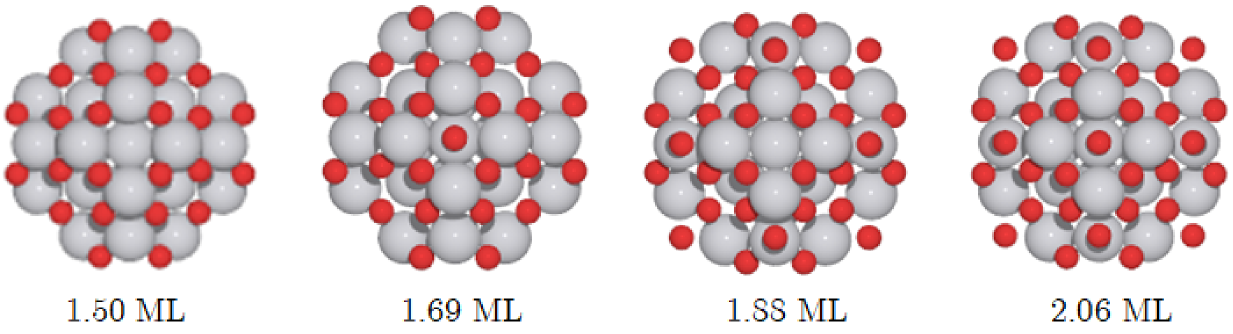

(a) Pt_{38}

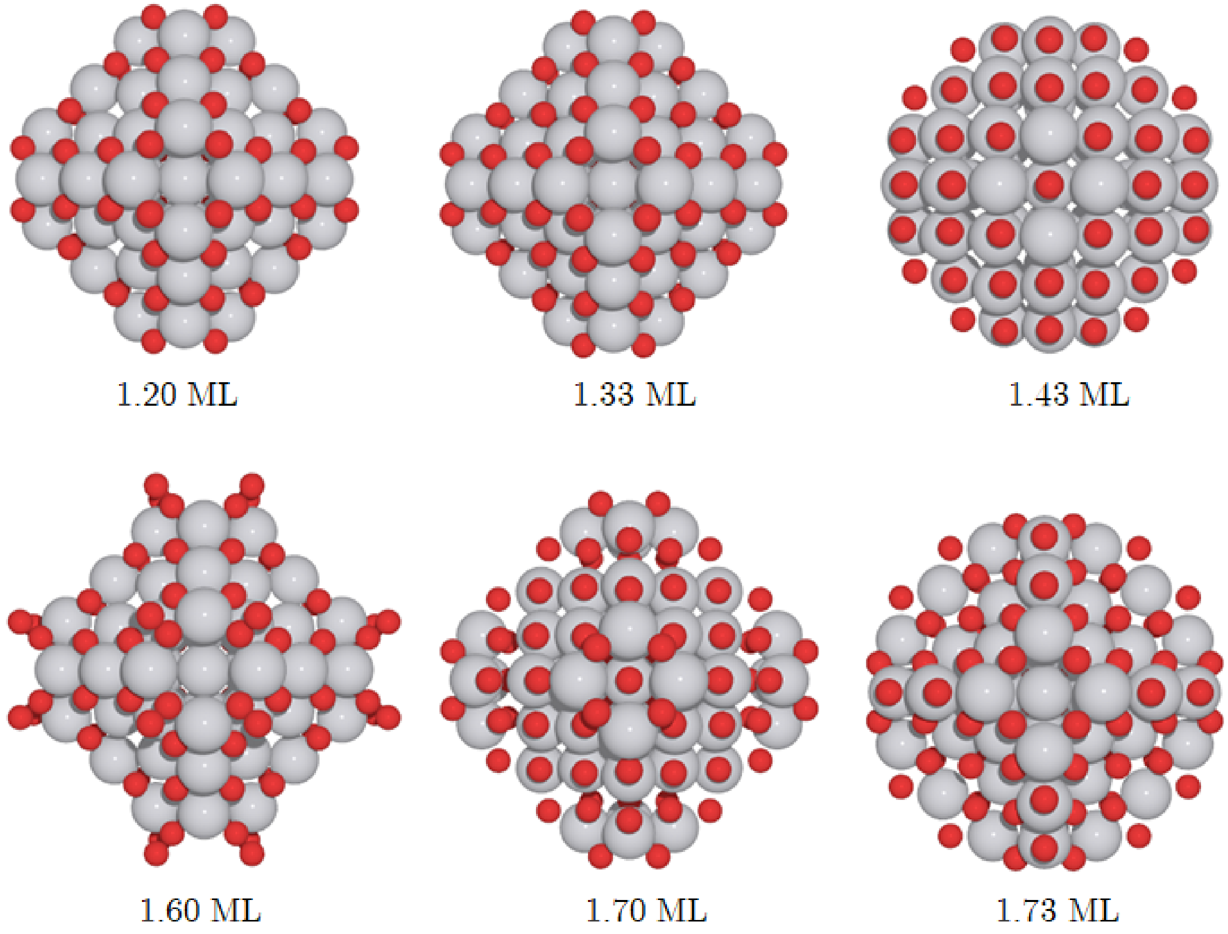

(b) Pt_{79}

Figure S2: Selected lowest-energy configurations at high oxygen coverage exhibiting higher degree of structural reorganization manifested by averaged Pt-Pt nearest-neighbor distance $\langle r_{\mathrm{Pt-Pt}} \rangle_{\mathrm{s}} > 3$ Å (see Table 7).

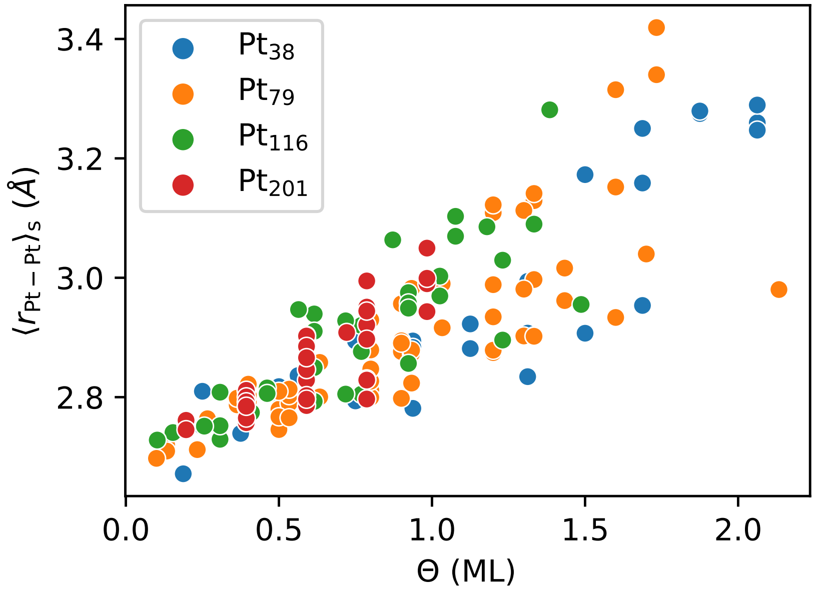

Figure S3: Dependence of the averaged nearest-neighbor distance between the surface platinum atoms $\langle r_{\mathrm{Pt-Pt}} \rangle_s$ on the oxygen coverage Θ.

Table S1: Structural properties of the computed configurations (see Table S2) for configuration descriptions: Coverage Θ (in monolayer, ML), adsorption energy per O atom $\overline{E}_{\mathrm{ads}}$ (in eV), averaged Pt-Pt nearest-neighbor distance for surface Pt atoms $\langle r_{\mathrm{Pt-Pt}} \rangle_s$ (in Å), averaged Pt-Pt nearest-neighbor distance for all Pt atoms $\langle r_{\mathrm{Pt-Pt}} \rangle$ (in Å), averaged Pt-O distance $\langle r_{\mathrm{Pt-O}} \rangle$ (in Å), averaged Pt-Pt coordination number $\langle \mathrm{CN}_{\mathrm{Pt-Pt}} \rangle$, averaged Pt-O coordination number $\langle \mathrm{CN}_{\mathrm{Pt-O}} \rangle$.

#	Formula	Θ (ML)	$\overline{E}_{\mathrm{ads}}$	$\langle r_{\mathrm{Pt-Pt}} \rangle_s$	$\langle r_{\mathrm{Pt-Pt}} \rangle$	$\langle r_{\mathrm{Pt-O}} \rangle$	$\langle \mathrm{CN}_{\mathrm{Pt-Pt}} \rangle$	$\langle \mathrm{CN}_{\mathrm{Pt-O}} \rangle$
1	$Pt_{38}O_1$	0.03	-1.69	2.68	2.73	2.06	7.4	0.1
2	$Pt_{38}O_1$	0.03	-1.61	2.68	2.73	1.94	7.6	0.1
3	$Pt_{38}O_1$	0.03	-1.58	2.68	2.73	2.06	7.4	0.1
4	$Pt_{38}O_1$	0.03	-1.27	2.68	2.73	2.23	7.6	0.1
5	$Pt_{38}O_1$	0.03	-1.23	2.71	2.73	1.93	7.6	0.1
6	$Pt_{38}O_1$	0.03	-1.03	2.68	2.73	1.77	7.6	0.0
7	$Pt_{38}O_1$	0.03	-0.47	2.68	2.73	1.77	7.4	0.0
8	$Pt_{38}O_6$	0.19	-1.36	2.67	2.75	2.25	7.6	0.6
9	$Pt_{38}O_8$	0.25	-1.69	2.75	2.71	2.06	6.3	0.6

Table S1: (continued)

#	Formula	Θ	$\overline{E}_{ads}$	$\langle r_{Pt-Pt} \rangle_s$	$\langle r_{Pt-Pt} \rangle$	$\langle r_{Pt-O} \rangle$	$\langle CN_{Pt-Pt} \rangle$	$\langle CN_{Pt-O} \rangle$
10	$Pt_{38}O_8$	0.25	-1.55	2.81	2.68	2.04	5.9	0.6
11	$Pt_{38}O_{12}$	0.38	-1.42	2.74	2.75	1.95	7.6	0.6
12	$Pt_{38}O_{16}$	0.50	-1.49	2.82	2.78	2.05	6.3	1.3
13	$Pt_{38}O_{16}$	0.50	-1.21	2.78	2.82	2.07	7.2	1.3
14	$Pt_{38}O_{18}$	0.56	-1.18	2.84	2.82	2.02	7.6	1.3
15	$Pt_{38}O_{24}$	0.75	-1.26	2.89	2.95	1.99	7.6	1.9
16	$Pt_{38}O_{24}$	0.75	-1.10	2.79	2.80	1.95	7.6	1.3
17	$Pt_{38}O_{24}$	0.75	-0.96	2.80	2.76	2.10	6.3	1.9
18	$Pt_{38}O_{30}$	0.94	-1.02	2.88	2.95	2.08	7.6	2.5
19	$Pt_{38}O_{30}$	0.94	-0.90	2.89	2.88	2.09	7.6	2.5
20	$Pt_{38}O_{30}$	0.94	-0.28	2.78	2.79	1.97	7.6	1.3
21	$Pt_{38}O_{36}$	1.12	-1.06	2.92	2.93	2.00	7.6	2.5
22	$Pt_{38}O_{36}$	1.12	-0.66	2.88	2.86	1.95	7.6	1.9
23	$Pt_{38}O_{36}$	1.12	-0.37	2.88	2.68	1.83	3.8	1.3
24	$Pt_{38}O_{42}$	1.31	-1.04	2.99	3.00	2.04	7.6	3.2
25	$Pt_{38}O_{42}$	1.31	-0.24	2.83	2.83	2.01	7.6	1.9
26	$Pt_{38}O_{42}$	1.31	0.11	2.91	2.89	2.09	7.6	2.5
27	$Pt_{38}O_{48}$	1.50	-1.20	3.17	3.06	1.98	6.3	3.2
28	$Pt_{38}O_{48}$	1.50	-1.19	3.17	3.06	1.98	6.3	3.2
29	$Pt_{38}O_{48}$	1.50	0.01	2.91	2.91	2.19	7.6	3.2
30	$Pt_{38}O_{54}$	1.69	-0.90	3.25	3.10	2.07	6.3	3.9
31	$Pt_{38}O_{54}$	1.69	-0.83	3.16	3.05	1.99	6.3	3.2
32	$Pt_{38}O_{54}$	1.69	0.20	2.95	2.94	2.12	7.6	3.2
33	$Pt_{38}O_{60}$	1.88	-0.68	3.28	2.98	2.02	5.1	3.8

Table S1: (continued)

#	Formula	Θ	$\overline{E}_{\mathrm{ads}}$	$\langle r_{\mathrm{Pt-Pt}}\rangle_{\mathrm{s}}$	$\langle r_{\mathrm{Pt-Pt}}\rangle$	$\langle r_{\mathrm{Pt-O}}\rangle$	$\langle \mathrm{CN}_{\mathrm{Pt-Pt}}\rangle$	$\langle \mathrm{CN}_{\mathrm{Pt-O}}\rangle$
34	$Pt_{38}O_{60}$	1.88	-0.53	3.28	3.02	2.01	5.1	3.8
35	$Pt_{38}O_{66}$	2.06	-0.51	3.25	3.07	2.08	6.3	4.6
36	$Pt_{38}O_{66}$	2.06	-0.26	3.29	3.03	2.03	5.1	3.8
37	$Pt_{38}O_{66}$	2.06	-0.15	3.26	2.79	2.04	3.2	3.8
38	$Pt_{79}O_{1}$	0.02	-1.48	2.70	2.75	1.94	8.5	0.0
39	$Pt_{79}O_{1}$	0.02	-1.47	2.70	2.75	1.93	8.5	0.0
40	$Pt_{79}O_{1}$	0.02	-1.40	2.70	2.75	2.05	8.5	0.0
41	$Pt_{79}O_{1}$	0.02	-1.40	2.70	2.75	2.01	8.5	0.0
42	$Pt_{79}O_{1}$	0.02	-1.34	2.70	2.75	2.05	8.5	0.0
43	$Pt_{79}O_{1}$	0.02	-1.32	2.70	2.75	2.24	8.5	0.1
44	$Pt_{79}O_{1}$	0.02	-1.22	2.70	2.75	2.04	8.4	0.0
45	$Pt_{79}O_{1}$	0.02	-0.92	2.70	2.75	1.77	8.5	0.0
46	$Pt_{79}O_{1}$	0.02	-0.43	2.70	2.75	1.79	8.5	0.0
47	$Pt_{79}O_{1}$	0.02	-0.12	2.70	2.75	1.80	8.5	0.0
48	$Pt_{79}O_{6}$	0.10	-1.18	2.70	2.75	2.25	8.5	0.3
49	$Pt_{79}O_{8}$	0.13	-1.40	2.72	2.77	2.04	8.5	0.3
50	$Pt_{79}O_{8}$	0.13	-1.33	2.71	2.80	2.00	8.5	0.3
51	$Pt_{79}O_{14}$	0.23	-1.07	2.71	2.80	2.12	8.5	0.6
52	$Pt_{79}O_{16}$	0.27	-1.33	2.76	2.77	2.03	7.9	0.6
53	$Pt_{79}O_{24}$	0.40	-1.40	2.78	2.82	2.01	8.5	0.9
54	$Pt_{79}O_{24}$	0.40	-1.28	2.79	2.79	1.93	8.5	0.6
55	$Pt_{79}O_{24}$	0.40	-1.25	2.82	2.82	2.03	8.5	0.9
56	$Pt_{79}O_{24}$	0.40	-1.18	2.77	2.78	2.17	8.5	0.9
57	$Pt_{79}O_{24}$	0.40	-1.12	2.80	2.81	2.07	8.5	0.9

Table S1: (continued)

#	Formula	Θ	$\overline{E}_{\text{ads}}$	$\langle r_{\text{Pt-Pt}} \rangle_{\text{s}}$	$\langle r_{\text{Pt-Pt}} \rangle$	$\langle r_{\text{Pt-O}} \rangle$	$\langle \text{CN}_{\text{Pt-Pt}} \rangle$	$\langle \text{CN}_{\text{Pt-O}} \rangle$
58	$Pt_{79}O_{24}$	0.40	-0.91	2.78	2.86	2.02	8.5	0.9
59	$Pt_{79}O_{30}$	0.50	-1.15	2.77	2.82	2.10	8.5	1.2
60	$Pt_{79}O_{30}$	0.50	-1.06	2.81	2.81	1.99	8.5	0.9
61	$Pt_{79}O_{30}$	0.50	-0.87	2.78	2.86	2.08	8.5	1.2
62	$Pt_{79}O_{30}$	0.50	-0.28	2.75	2.77	1.98	8.5	0.6
63	$Pt_{79}O_{32}$	0.53	-1.17	2.80	2.81	1.97	8.5	0.9
64	$Pt_{79}O_{32}$	0.53	-1.09	2.77	2.79	1.99	8.5	0.9
65	$Pt_{79}O_{32}$	0.53	-1.03	2.81	2.83	2.03	8.5	1.2
66	$Pt_{79}O_{32}$	0.53	-0.20	2.79	2.73	1.90	6.4	0.6
67	$Pt_{79}O_{38}$	0.63	-1.03	2.86	2.84	1.99	8.5	1.2
68	$Pt_{79}O_{38}$	0.63	-0.86	2.80	2.83	2.09	8.5	1.5
69	$Pt_{79}O_{48}$	0.80	-1.04	2.93	2.85	2.08	8.2	1.8
70	$Pt_{79}O_{48}$	0.80	-1.03	2.85	2.84	2.00	8.5	1.5
71	$Pt_{79}O_{48}$	0.80	-0.95	2.83	2.84	2.01	8.5	1.5
72	$Pt_{79}O_{48}$	0.80	-0.93	2.84	2.88	2.00	8.5	1.5
73	$Pt_{79}O_{48}$	0.80	-0.91	2.88	2.91	2.02	8.5	1.8
74	$Pt_{79}O_{48}$	0.80	-0.74	2.81	2.80	1.96	8.5	1.2
75	$Pt_{79}O_{48}$	0.80	-0.14	2.80	2.83	2.14	8.5	1.5
76	$Pt_{79}O_{54}$	0.90	-1.11	2.96	2.91	2.06	8.5	2.1
77	$Pt_{79}O_{54}$	0.90	-1.02	2.89	2.87	2.03	8.5	1.8
78	$Pt_{79}O_{54}$	0.90	-0.88	2.89	2.90	2.02	7.9	1.8
79	$Pt_{79}O_{54}$	0.90	-0.80	2.88	2.92	2.06	8.5	2.1
80	$Pt_{79}O_{54}$	0.90	-0.36	2.80	2.79	1.99	8.5	1.2
81	$Pt_{79}O_{56}$	0.93	-0.97	2.88	2.86	2.01	8.5	1.8

Table S1: (continued)

#	Formula	Θ	$\overline{E}_{\text{ads}}$	$\langle r_{\text{Pt-Pt}}\rangle_{\text{s}}$	$\langle r_{\text{Pt-Pt}}\rangle$	$\langle r_{\text{Pt-O}}\rangle$	$\langle \text{CN}_{\text{Pt-Pt}}\rangle$	$\langle \text{CN}_{\text{Pt-O}}\rangle$
82	$Pt_{79}O_{56}$	0.93	-0.93	2.98	2.89	2.07	8.2	2.1
83	$Pt_{79}O_{56}$	0.93	-0.79	2.82	2.81	1.98	8.5	1.5
84	$Pt_{79}O_{56}$	0.93	-0.58	2.87	2.77	2.00	5.5	1.8
85	$Pt_{79}O_{62}$	1.03	-1.00	2.99	2.92	2.06	8.2	2.4
86	$Pt_{79}O_{62}$	1.03	-0.98	2.92	2.89	2.04	8.5	2.1
87	$Pt_{79}O_{72}$	1.20	-1.22	3.12	2.92	1.99	6.7	2.4
88	$Pt_{79}O_{72}$	1.20	-1.22	3.12	2.92	1.99	6.7	2.4
89	$Pt_{79}O_{72}$	1.20	-0.74	2.93	2.92	2.02	8.5	2.4
90	$Pt_{79}O_{72}$	1.20	-0.73	2.99	2.92	2.03	8.5	2.1
91	$Pt_{79}O_{72}$	1.20	-0.73	3.11	2.78	2.01	6.1	2.1
92	$Pt_{79}O_{72}$	1.20	-0.68	2.87	2.87	2.00	8.5	2.1
93	$Pt_{79}O_{72}$	1.20	-0.27	2.88	2.91	2.02	8.5	1.8
94	$Pt_{79}O_{78}$	1.30	-0.96	3.11	2.98	2.00	7.9	2.4
95	$Pt_{79}O_{78}$	1.30	-0.76	2.98	2.95	2.04	8.5	2.7
96	$Pt_{79}O_{78}$	1.30	-0.40	2.90	2.79	2.09	6.4	2.4
97	$Pt_{79}O_{80}$	1.33	-1.11	3.14	3.00	2.01	7.9	2.7
98	$Pt_{79}O_{80}$	1.33	-0.67	3.00	2.93	2.04	8.5	2.4
99	$Pt_{79}O_{80}$	1.33	-0.67	3.13	2.79	2.03	6.1	2.4
100	$Pt_{79}O_{80}$	1.33	-0.62	2.90	2.84	1.98	7.0	2.1
101	$Pt_{79}O_{80}$	1.33	-0.62	2.90	2.84	1.98	7.0	2.1
102	$Pt_{79}O_{86}$	1.43	-0.66	3.02	2.99	2.09	6.7	3.3
103	$Pt_{79}O_{86}$	1.43	-0.37	2.96	2.83	2.09	6.4	2.7
104	$Pt_{79}O_{96}$	1.60	-0.62	3.15	2.93	2.02	6.7	2.4
105	$Pt_{79}O_{96}$	1.60	-0.60	3.32	2.77	2.02	4.6	3.0

Table S1: (continued)

#	Formula	Θ	$\overline{E}_{\text{ads}}$	$\langle r_{\text{Pt-Pt}} \rangle_{\text{s}}$	$\langle r_{\text{Pt-Pt}} \rangle$	$\langle r_{\text{Pt-O}} \rangle$	$\langle \text{CN}_{\text{Pt-Pt}} \rangle$	$\langle \text{CN}_{\text{Pt-O}} \rangle$
106	$Pt_{79}O_{96}$	1.60	-0.30	2.93	2.92	2.02	8.5	2.4
107	$Pt_{79}O_{102}$	1.70	-0.49	3.04	2.88	2.07	6.1	3.4
108	$Pt_{79}O_{104}$	1.73	-0.52	3.34	2.79	2.03	4.6	3.3
109	$Pt_{79}O_{104}$	1.73	-0.44	3.42	2.82	1.96	3.9	2.7
110	$Pt_{79}O_{128}$	2.13	-0.12	2.98	2.87	1.96	7.3	1.5
111	$Pt_{116}O_{1}$	0.01	-1.63	2.72	2.75	1.93	8.9	0.0
112	$Pt_{116}O_{1}$	0.01	-1.49	2.72	2.75	1.95	8.9	0.0
113	$Pt_{116}O_{1}$	0.01	-1.40	2.72	2.75	2.09	8.9	0.0
114	$Pt_{116}O_{1}$	0.01	-1.39	2.72	2.75	2.07	8.9	0.0
115	$Pt_{116}O_{1}$	0.01	-1.33	2.72	2.75	1.94	8.9	0.0
116	$Pt_{116}O_{1}$	0.01	-1.24	2.72	2.75	2.04	8.9	0.0
117	$Pt_{116}O_{1}$	0.01	-1.23	2.72	2.76	2.04	8.9	0.0
118	$Pt_{116}O_{1}$	0.01	-1.01	2.72	2.76	2.02	8.9	0.0
119	$Pt_{116}O_{1}$	0.01	-0.87	2.72	2.75	1.77	8.9	0.0
120	$Pt_{116}O_{1}$	0.01	-0.56	2.72	2.75	1.78	8.9	0.0
121	$Pt_{116}O_{1}$	0.01	-0.25	2.71	2.75	1.81	8.9	0.0
122	$Pt_{116}O_{8}$	0.10	-1.04	2.73	2.79	2.01	8.9	0.2
123	$Pt_{116}O_{12}$	0.15	-1.47	2.74	2.76	1.95	8.9	0.2
124	$Pt_{116}O_{20}$	0.26	-1.07	2.75	2.77	2.00	8.9	0.4
125	$Pt_{116}O_{24}$	0.31	-1.08	2.75	2.78	2.09	8.9	0.6
126	$Pt_{116}O_{24}$	0.31	-0.96	2.81	2.81	2.05	8.9	0.6
127	$Pt_{116}O_{24}$	0.31	-0.86	2.73	2.76	2.23	8.5	0.8
128	$Pt_{116}O_{32}$	0.41	-0.76	2.77	2.79	2.08	8.9	0.8
129	$Pt_{116}O_{36}$	0.46	-1.02	2.81	2.79	2.09	8.9	0.8

Table S1: (continued)

#	Formula	Θ	$\overline{E}_{\mathrm{ads}}$	$\langle r_{\mathrm{Pt-Pt}}\rangle_{\mathrm{s}}$	$\langle r_{\mathrm{Pt-Pt}}\rangle$	$\langle r_{\mathrm{Pt-O}}\rangle$	$\langle \mathrm{CN}_{\mathrm{Pt-Pt}}\rangle$	$\langle \mathrm{CN}_{\mathrm{Pt-O}}\rangle$
130	$Pt_{116}O_{36}$	0.46	-1.01	2.82	2.81	2.03	8.9	0.8
131	$Pt_{116}O_{44}$	0.56	-0.94	2.95	2.83	2.05	8.9	1.0
132	$Pt_{116}O_{48}$	0.62	-1.07	2.79	2.80	1.95	8.9	0.8
133	$Pt_{116}O_{48}$	0.62	-0.98	2.91	2.80	2.04	7.4	1.2
134	$Pt_{116}O_{48}$	0.62	-0.97	2.85	2.85	2.04	8.9	1.2
135	$Pt_{116}O_{48}$	0.62	-0.79	2.94	2.82	2.12	8.1	1.2
136	$Pt_{116}O_{48}$	0.62	-0.71	2.85	2.83	2.07	8.9	1.2
137	$Pt_{116}O_{56}$	0.72	-1.00	2.81	2.81	1.97	8.9	1.0
138	$Pt_{116}O_{56}$	0.72	-0.85	2.93	2.82	2.04	7.4	1.4
139	$Pt_{116}O_{60}$	0.77	-0.94	2.88	2.86	2.03	8.9	1.4
140	$Pt_{116}O_{60}$	0.77	-0.87	2.80	2.80	1.96	8.9	1.0
141	$Pt_{116}O_{60}$	0.77	-0.87	2.92	2.86	2.05	8.7	1.4
142	$Pt_{116}O_{68}$	0.87	-0.88	3.06	2.84	1.95	7.7	1.2
143	$Pt_{116}O_{72}$	0.92	-1.25	2.96	2.88	1.97	8.9	1.4
144	$Pt_{116}O_{72}$	0.92	-1.01	2.95	2.86	2.07	8.1	1.9
145	$Pt_{116}O_{72}$	0.92	-0.80	2.86	2.84	2.00	8.9	1.4
146	$Pt_{116}O_{72}$	0.92	-0.73	2.98	2.88	2.08	8.7	1.9
147	$Pt_{116}O_{80}$	1.03	-1.13	2.97	2.88	1.99	8.9	1.7
148	$Pt_{116}O_{80}$	1.03	-0.85	3.00	2.84	2.06	7.2	2.1
149	$Pt_{116}O_{84}$	1.08	-0.96	3.07	2.94	2.04	8.5	2.1
150	$Pt_{116}O_{84}$	1.08	-0.82	3.10	2.86	1.98	7.4	1.7
151	$Pt_{116}O_{92}$	1.18	-0.88	3.09	2.98	2.04	8.9	2.3
152	$Pt_{116}O_{96}$	1.23	-0.96	3.03	2.92	2.01	8.9	2.1
153	$Pt_{116}O_{96}$	1.23	-0.57	2.90	2.82	2.03	7.2	1.7

Table S1: (continued)

#	Formula	Θ	$\overline{E}_{\mathrm{ads}}$	$\langle r_{\mathrm{Pt-Pt}} \rangle_{\mathrm{s}}$	$\langle r_{\mathrm{Pt-Pt}} \rangle$	$\langle r_{\mathrm{Pt-O}} \rangle$	$\langle \mathrm{CN}_{\mathrm{Pt-Pt}} \rangle$	$\langle \mathrm{CN}_{\mathrm{Pt-O}} \rangle$
154	$Pt_{116}O_{104}$	1.33	-0.58	3.09	2.75	1.96	6.0	1.4
155	$Pt_{116}O_{108}$	1.38	-0.73	3.28	2.79	1.99	5.4	2.3
156	$Pt_{116}O_{116}$	1.49	-0.44	2.96	2.86	2.03	7.2	2.1
157	$Pt_{201}O_{1}$	0.01	-1.53	2.72	2.77	1.93	9.4	0.0
158	$Pt_{201}O_{1}$	0.01	-1.31	2.72	2.77	1.94	9.4	0.0
159	$Pt_{201}O_{1}$	0.01	-1.31	2.72	2.77	2.06	9.4	0.0
160	$Pt_{201}O_{1}$	0.01	-1.22	2.71	2.77	1.95	9.4	0.0
161	$Pt_{201}O_{1}$	0.01	-1.18	2.72	2.77	2.10	9.4	0.0
162	$Pt_{201}O_{1}$	0.01	-1.17	2.72	2.77	2.05	9.4	0.0
163	$Pt_{201}O_{1}$	0.01	-1.11	2.72	2.77	2.02	9.4	0.0
164	$Pt_{201}O_{1}$	0.01	-1.08	2.72	2.77	2.04	9.4	0.0
165	$Pt_{201}O_{1}$	0.01	-0.99	2.72	2.77	2.06	9.4	0.0
166	$Pt_{201}O_{1}$	0.01	-0.83	2.72	2.77	2.02	9.4	0.0
167	$Pt_{201}O_{1}$	0.01	-0.80	2.71	2.77	1.77	9.4	0.0
168	$Pt_{201}O_{1}$	0.01	-0.56	2.72	2.77	1.79	9.4	0.0
169	$Pt_{201}O_{1}$	0.01	-0.54	2.71	2.77	1.79	9.4	0.0
170	$Pt_{201}O_{1}$	0.01	-0.07	2.71	2.77	1.81	9.4	0.0
171	$Pt_{201}O_{1}$	0.01	0.15	2.71	2.77	1.81	9.4	0.0
172	$Pt_{201}O_{24}$	0.20	-1.23	2.76	2.78	1.94	9.4	0.2
173	$Pt_{201}O_{24}$	0.20	-1.02	2.75	2.80	2.02	9.4	0.4
174	$Pt_{201}O_{24}$	0.20	-0.90	2.75	2.78	2.05	9.4	0.4
175	$Pt_{201}O_{48}$	0.39	-1.28	2.81	2.81	2.00	9.4	0.6
176	$Pt_{201}O_{48}$	0.39	-1.19	2.81	2.81	2.06	9.4	0.7
177	$Pt_{201}O_{48}$	0.39	-1.08	2.80	2.79	2.02	9.4	0.6

Table S1: (continued)

#	Formula	Θ	$\overline{E}_{\text{ads}}$	$\langle r_{\text{Pt-Pt}} \rangle_{\text{s}}$	$\langle r_{\text{Pt-Pt}} \rangle$	$\langle r_{\text{Pt-O}} \rangle$	$\langle \text{CN}_{\text{Pt-Pt}} \rangle$	$\langle \text{CN}_{\text{Pt-O}} \rangle$
178	$Pt_{201}O_{48}$	0.39	-1.06	2.79	2.80	1.99	9.4	0.6
179	$Pt_{201}O_{48}$	0.39	-1.04	2.77	2.78	1.95	9.4	0.5
180	$Pt_{201}O_{48}$	0.39	-0.99	2.78	2.81	2.04	9.4	0.7
181	$Pt_{201}O_{48}$	0.39	-0.90	2.78	2.79	2.07	9.4	0.6
182	$Pt_{201}O_{48}$	0.39	-0.76	2.76	2.80	2.12	9.4	0.7
183	$Pt_{201}O_{72}$	0.59	-1.04	2.90	2.83	2.04	8.7	1.1
184	$Pt_{201}O_{72}$	0.59	-0.98	2.89	2.82	2.03	9.1	1.0
185	$Pt_{201}O_{72}$	0.59	-0.95	2.87	2.83	2.05	9.3	1.1
186	$Pt_{201}O_{72}$	0.59	-0.93	2.80	2.80	2.00	9.4	0.8
187	$Pt_{201}O_{72}$	0.59	-0.93	2.89	2.82	2.04	9.4	1.0
188	$Pt_{201}O_{72}$	0.59	-0.92	2.80	2.81	2.00	9.4	0.8
189	$Pt_{201}O_{72}$	0.59	-0.91	2.83	2.83	2.01	9.4	1.0
190	$Pt_{201}O_{72}$	0.59	-0.90	2.85	2.82	2.10	9.4	1.1
191	$Pt_{201}O_{72}$	0.59	-0.84	2.90	2.82	2.04	9.2	1.0
192	$Pt_{201}O_{72}$	0.59	-0.83	2.80	2.79	1.96	9.4	0.7
193	$Pt_{201}O_{72}$	0.59	-0.73	2.90	2.82	2.08	9.0	1.1
194	$Pt_{201}O_{72}$	0.59	-0.38	2.79	2.78	1.92	8.7	0.5
195	$Pt_{201}O_{88}$	0.72	-1.08	2.91	2.85	2.02	9.4	1.2
196	$Pt_{201}O_{96}$	0.79	-1.20	2.94	2.87	1.98	9.4	1.2
197	$Pt_{201}O_{96}$	0.79	-0.95	2.95	2.88	2.02	9.4	1.3
198	$Pt_{201}O_{96}$	0.79	-0.92	2.99	2.82	1.98	8.7	1.1
199	$Pt_{201}O_{96}$	0.79	-0.87	2.92	2.86	2.07	9.3	1.4
200	$Pt_{201}O_{96}$	0.79	-0.84	2.83	2.81	1.99	9.4	1.1
201	$Pt_{201}O_{96}$	0.79	-0.79	2.83	2.82	1.99	9.4	1.1

Table S1: (continued)

#	Formula	Θ	$\overline{E}_{\mathrm{ads}}$	$\langle r_{\mathrm{Pt-Pt}} \rangle_{\mathrm{s}}$	$\langle r_{\mathrm{Pt-Pt}} \rangle$	$\langle r_{\mathrm{Pt-O}} \rangle$	$\langle \mathrm{CN}_{\mathrm{Pt-Pt}} \rangle$	$\langle \mathrm{CN}_{\mathrm{Pt-O}} \rangle$
202	$Pt_{201}O_{96}$	0.79	-0.78	2.90	2.85	2.09	9.4	1.4
203	$Pt_{201}O_{96}$	0.79	-0.38	2.80	2.79	1.96	9.4	0.7
204	$Pt_{201}O_{120}$	0.98	-1.11	2.99	2.87	2.00	9.1	1.6
205	$Pt_{201}O_{120}$	0.98	-1.03	3.00	2.88	2.01	9.2	1.6
206	$Pt_{201}O_{120}$	0.98	-0.84	3.05	2.87	2.07	8.8	1.8
207	$Pt_{201}O_{120}$	0.98	-0.75	2.94	2.86	1.99	9.4	1.2

Table S2: Full list of the computed configurations specified by the number of oxygen atoms N_s of the specified site, coordination number CN and generalized coordination number $\overline{\mathrm{CN}}$. The list ordering is the same as in Table S1.

#	Formula	Configuration (N_s/site type/CN/$\overline{\mathrm{CN}}$)
1	$Pt_{38}O_1$	1/hcp/11/4.09
2	$Pt_{38}O_1$	1/bridge/8/3.5
3	$Pt_{38}O_1$	1/fcc/10/3.68
4	$Pt_{38}O_1$	1/trough/9/2.77
5	$Pt_{38}O_1$	1/bridge/8/3.67
6	$Pt_{38}O_1$	1/ontop/6/4.0
7	$Pt_{38}O_1$	1/ontop/9/6.0
8	$Pt_{38}O_6$	6/trough/9/2.77
9	$Pt_{38}O_8$	8/hcp/11/4.09
10	$Pt_{38}O_8$	8/fcc/10/3.68
11	$Pt_{38}O_{12}$	12/bridge/8/3.67
12	$Pt_{38}O_{16}$	16/hcp/11/4.09
13	$Pt_{38}O_{16}$	16/fcc/10/3.68
14	$Pt_{38}O_{18}$	6/trough/9/2.77, 12/bridge/8/3.67
15	$Pt_{38}O_{24}$	24/hcp/11/4.09
16	$Pt_{38}O_{24}$	24/bridge/8/3.5

Table S2: (continued)

#	Formula	Configuration (N_s/Type/CN/$\overline{\mathrm{CN}}$)
17	$Pt_{38}O_{24}$	24/fcc/10/3.68
18	$Pt_{38}O_{30}$	24/hcp/11/4.09, 6/trough/9/2.77
19	$Pt_{38}O_{30}$	24/fcc/10/3.68, 6/trough/9/2.77
20	$Pt_{38}O_{30}$	24/bridge/8/3.5, 6/trough/9/2.77
21	$Pt_{38}O_{36}$	24/hcp/11/4.09, 12/bridge/8/3.67
22	$Pt_{38}O_{36}$	24/bridge/8/3.5, 12/bridge/8/3.67
23	$Pt_{38}O_{36}$	24/fcc/10/3.68, 12/bridge/8/3.67
24	$Pt_{38}O_{42}$	24/hcp/11/4.09, 6/trough/9/2.77, 12/bridge/8/3.67
25	$Pt_{38}O_{42}$	24/bridge/8/3.5, 6/trough/9/2.77, 12/bridge/8/3.67
26	$Pt_{38}O_{42}$	24/fcc/10/3.68, 6/trough/9/2.77, 12/bridge/8/3.67
27	$Pt_{38}O_{48}$	24/hcp/11/4.09, 24/fcc/10/3.68
28	$Pt_{38}O_{48}$	24/bridge/8/3.5, 24/fcc/10/3.68
29	$Pt_{38}O_{48}$	24/hcp/11/4.09, 24/bridge/8/3.5
30	$Pt_{38}O_{54}$	24/hcp/11/4.09, 24/fcc/10/3.68, 6/trough/9/2.77
31	$Pt_{38}O_{54}$	24/bridge/8/3.5, 24/fcc/10/3.68, 6/trough/9/2.77
32	$Pt_{38}O_{54}$	24/hcp/11/4.09, 24/bridge/8/3.5, 6/trough/9/2.77
33	$Pt_{38}O_{60}$	24/hcp/11/4.09, 24/fcc/10/3.68, 12/bridge/8/3.67

Table S2: (continued)

#	Formula	Configuration (N_s/Type/CN/$\overline{\text{CN}}$)
34	$Pt_{38}O_{60}$	24/bridge/8/3.5, 24/fcc/10/3.68, 12/bridge/8/3.67
35	$Pt_{38}O_{66}$	24/hcp/11/4.09, 24/fcc/10/3.68, 6/trough/9/2.77, 12/bridge/8/3.67
36	$Pt_{38}O_{66}$	24/bridge/8/3.5, 24/fcc/10/3.68, 6/trough/9/2.77, 12/bridge/8/3.67
37	$Pt_{38}O_{66}$	24/hcp/11/4.09, 24/bridge/8/3.5, 6/trough/9/2.77, 12/bridge/8/3.67
38	$Pt_{79}O_1$	1/bridge/8/3.61
39	$Pt_{79}O_1$	1/bridge/9/4.33
40	$Pt_{79}O_1$	1/fcc/11/4.41
41	$Pt_{79}O_1$	1/fcc/15/5.86
42	$Pt_{79}O_1$	1/hcp/11/4.45
43	$Pt_{79}O_1$	1/trough/9/2.92
44	$Pt_{79}O_1$	1/hcp/14/5.68
45	$Pt_{79}O_1$	1/ontop/6/4.08
46	$Pt_{79}O_1$	1/ontop/7/5.0
47	$Pt_{79}O_1$	1/ontop/9/6.67
48	$Pt_{79}O_6$	6/trough/9/2.92
49	$Pt_{79}O_8$	8/fcc/11/4.41
50	$Pt_{79}O_8$	8/fcc/15/5.86

Table S2: (continued)

#	Formula	Configuration (N_s/Type/CN/$\overline{\mathrm{CN}}$)
51	$Pt_{79}O_{14}$	8/fcc/15/5.86, 6/trough/9/2.92
52	$Pt_{79}O_{16}$	16/fcc/11/4.41
53	$Pt_{79}O_{24}$	24/hcp/11/4.45
54	$Pt_{79}O_{24}$	24/bridge/9/4.33
55	$Pt_{79}O_{24}$	24/fcc/11/4.41
56	$Pt_{79}O_{24}$	24/bridge/8/3.61
57	$Pt_{79}O_{24}$	24/fcc/11/4.41
58	$Pt_{79}O_{24}$	24/hcp/14/5.68
59	$Pt_{79}O_{30}$	24/hcp/11/4.45, 6/trough/9/2.92
60	$Pt_{79}O_{30}$	24/bridge/9/4.33, 6/trough/9/2.92
61	$Pt_{79}O_{30}$	6/trough/9/2.92, 24/hcp/14/5.68
62	$Pt_{79}O_{30}$	24/bridge/8/3.61, 6/trough/9/2.92
63	$Pt_{79}O_{32}$	24/bridge/9/4.33, 8/fcc/15/5.86
64	$Pt_{79}O_{32}$	24/bridge/8/3.61, 8/fcc/15/5.86
65	$Pt_{79}O_{32}$	8/fcc/15/5.86, 24/hcp/11/4.45
66	$Pt_{79}O_{32}$	8/fcc/15/5.86, 24/hcp/14/5.68
67	$Pt_{79}O_{38}$	24/bridge/9/4.33, 8/fcc/15/5.86, 6/trough/9/2.92

Table S2: (continued)

#	Formula	Configuration (N_s/Type/CN/$\overline{\mathrm{CN}}$)
68	$Pt_{79}O_{38}$	8/fcc/15/5.86, 24/hcp/11/4.45, 6/trough/9/2.92
69	$Pt_{79}O_{48}$	48/fcc/11/4.41
70	$Pt_{79}O_{48}$	24/bridge/9/4.33, 24/hcp/11/4.45
71	$Pt_{79}O_{48}$	24/bridge/8/3.61, 24/hcp/14/5.68
72	$Pt_{79}O_{48}$	24/bridge/9/4.33, 24/hcp/14/5.68
73	$Pt_{79}O_{48}$	24/hcp/11/4.45, 24/hcp/14/5.68
74	$Pt_{79}O_{48}$	24/bridge/8/3.61, 24/bridge/9/4.33
75	$Pt_{79}O_{48}$	24/bridge/8/3.61, 24/hcp/11/4.45
76	$Pt_{79}O_{54}$	48/fcc/11/4.41, 6/trough/9/2.92
77	$Pt_{79}O_{54}$	24/bridge/9/4.33, 24/hcp/11/4.45, 6/trough/9/2.92
78	$Pt_{79}O_{54}$	24/bridge/9/4.33, 6/trough/9/2.92, 24/hcp/14/5.68
79	$Pt_{79}O_{54}$	24/hcp/11/4.45, 6/trough/9/2.92, 24/hcp/14/5.68
80	$Pt_{79}O_{54}$	24/bridge/8/3.61, 24/bridge/9/4.33, 6/trough/9/2.92
81	$Pt_{79}O_{56}$	24/bridge/9/4.33, 8/fcc/15/5.86, 24/hcp/11/4.45
82	$Pt_{79}O_{56}$	48/fcc/11/4.41, 8/fcc/15/5.86
83	$Pt_{79}O_{56}$	24/bridge/8/3.61, 24/bridge/9/4.33, 8/fcc/15/5.86
84	$Pt_{79}O_{56}$	24/bridge/8/3.61, 8/fcc/15/5.86, 24/hcp/11/4.45

Table S2: (continued)

#	Formula	Configuration (N_s/Type/CN/$\overline{\mathrm{CN}}$)
85	$Pt_{79}O_{62}$	48/fcc/11/4.41, 8/fcc/15/5.86, 6/trough/9/2.92
86	$Pt_{79}O_{62}$	24/bridge/9/4.33, 8/fcc/15/5.86, 24/hcp/11/4.45, 6/trough/9/2.92
87	$Pt_{79}O_{72}$	24/bridge/8/3.61, 48/fcc/11/4.41
88	$Pt_{79}O_{72}$	48/fcc/11/4.41, 24/hcp/11/4.45
89	$Pt_{79}O_{72}$	24/bridge/9/4.33, 24/hcp/11/4.45, 24/hcp/14/5.68
90	$Pt_{79}O_{72}$	24/bridge/9/4.33, 48/fcc/11/4.41
91	$Pt_{79}O_{72}$	24/bridge/8/3.61, 24/bridge/9/4.33, 24/hcp/11/4.45
92	$Pt_{79}O_{72}$	24/bridge/8/3.61, 24/bridge/9/4.33, 24/hcp/14/5.68
93	$Pt_{79}O_{72}$	24/bridge/8/3.61, 24/hcp/11/4.45, 24/hcp/14/5.68
94	$Pt_{79}O_{78}$	24/bridge/8/3.61, 48/fcc/11/4.41, 6/trough/9/2.92
95	$Pt_{79}O_{78}$	24/bridge/9/4.33, 24/hcp/11/4.45, 6/trough/9/2.92, 24/hcp/14/5.68
96	$Pt_{79}O_{78}$	24/bridge/9/4.33, 48/fcc/11/4.41, 6/trough/9/2.92
97	$Pt_{79}O_{80}$	24/bridge/8/3.61, 48/fcc/11/4.41, 8/fcc/15/5.86
98	$Pt_{79}O_{80}$	24/bridge/9/4.33, 48/fcc/11/4.41, 8/fcc/15/5.86
99	$Pt_{79}O_{80}$	24/bridge/8/3.61, 24/bridge/9/4.33, 8/fcc/15/5.86, 24/hcp/11/4.45
100	$Pt_{79}O_{80}$	24/bridge/8/3.61, 24/bridge/9/4.33, 8/fcc/15/5.86, 24/hcp/14/5.68
101	$Pt_{79}O_{80}$	24/bridge/9/4.33, 8/fcc/15/5.86, 24/hcp/11/4.45, 24/hcp/14/5.68

Table S2: (continued)

#	Formula	Configuration (N_s/Type/CN/$\overline{\mathrm{CN}}$)
102	$Pt_{79}O_{86}$	24/bridge/9/4.33, 8/fcc/15/5.86, 24/hcp/11/4.45, 6/trough/9/2.92, 24/hcp/14/5.68
103	$Pt_{79}O_{86}$	24/bridge/9/4.33, 48/fcc/11/4.41, 8/fcc/15/5.86, 6/trough/9/2.92
104	$Pt_{79}O_{96}$	24/bridge/8/3.61, 48/fcc/11/4.41, 24/hcp/11/4.45
105	$Pt_{79}O_{96}$	24/bridge/8/3.61, 24/bridge/9/4.33, 48/fcc/11/4.41
106	$Pt_{79}O_{96}$	24/bridge/8/3.61, 24/bridge/9/4.33, 24/hcp/11/4.45, 24/hcp/14/5.68
107	$Pt_{79}O_{102}$	24/bridge/8/3.61, 24/bridge/9/4.33, 24/hcp/11/4.45, 6/trough/9/2.92, 24/hcp/14/5.68
108	$Pt_{79}O_{104}$	24/bridge/8/3.61, 24/bridge/9/4.33, 48/fcc/11/4.41, 8/fcc/15/5.86
109	$Pt_{79}O_{104}$	24/bridge/8/3.61, 24/bridge/9/4.33, 8/fcc/15/5.86, 24/hcp/11/4.45, 24/hcp/14/5.68
110	$Pt_{79}O_{128}$	24/bridge/8/3.61, 24/bridge/9/4.33, 48/fcc/11/4.41, 8/fcc/15/5.86, 24/hcp/11/4.45
111	$Pt_{116}O_1$	1/bridge/9/4.33
112	$Pt_{116}O_1$	1/bridge/8/3.89
113	$Pt_{116}O_1$	1/trough/13/4.46
114	$Pt_{116}O_1$	1/fcc/10/4.14
115	$Pt_{116}O_1$	1/bridge/11/5.5
116	$Pt_{116}O_1$	1/hcp/12/4.95
117	$Pt_{116}O_1$	1/fcc/13/5.23
118	$Pt_{116}O_1$	1/hcp/16/6.41

Table S2: (continued)

#	Formula	Configuration (N_s/Type/CN/$\overline{\text{CN}}$)
119	$Pt_{116}O_1$	1/ontop/6/4.17
120	$Pt_{116}O_1$	1/ontop/7/5.17
121	$Pt_{116}O_1$	1/ontop/8/6.33
122	$Pt_{116}O_8$	8/hcp/16/6.41
123	$Pt_{116}O_{12}$	12/bridge/8/3.89
124	$Pt_{116}O_{20}$	12/bridge/8/3.89, 8/hcp/16/6.41
125	$Pt_{116}O_{24}$	24/fcc/10/4.14
126	$Pt_{116}O_{24}$	24/fcc/13/5.23
127	$Pt_{116}O_{24}$	24/trough/13/4.46
128	$Pt_{116}O_{32}$	24/fcc/10/4.14, 8/hcp/16/6.41
129	$Pt_{116}O_{36}$	12/bridge/8/3.89, 24/trough/13/4.46
130	$Pt_{116}O_{36}$	12/bridge/8/3.89, 24/fcc/13/5.23
131	$Pt_{116}O_{44}$	12/bridge/8/3.89, 24/trough/13/4.46, 8/hcp/16/6.41
132	$Pt_{116}O_{48}$	48/bridge/9/4.33
133	$Pt_{116}O_{48}$	24/trough/13/4.46, 24/fcc/10/4.14
134	$Pt_{116}O_{48}$	48/hcp/12/4.95
135	$Pt_{116}O_{48}$	24/trough/13/4.46, 24/fcc/13/5.23

Table S2: (continued)

#	Formula	Configuration (N_s/Type/CN/$\overline{\mathrm{CN}}$)
136	$Pt_{116}O_{48}$	24/fcc/10/4.14, 24/fcc/13/5.23
137	$Pt_{116}O_{56}$	48/bridge/9/4.33, 8/hcp/16/6.41
138	$Pt_{116}O_{56}$	24/trough/13/4.46, 24/fcc/10/4.14, 8/hcp/16/6.41
139	$Pt_{116}O_{60}$	12/bridge/8/3.89, 48/hcp/12/4.95
140	$Pt_{116}O_{60}$	48/bridge/9/4.33, 12/bridge/8/3.89
141	$Pt_{116}O_{60}$	12/bridge/8/3.89, 24/trough/13/4.46, 24/fcc/13/5.23
142	$Pt_{116}O_{68}$	48/bridge/9/4.33, 12/bridge/8/3.89, 8/hcp/16/6.41
143	$Pt_{116}O_{72}$	48/bridge/9/4.33, 24/fcc/10/4.14
144	$Pt_{116}O_{72}$	24/trough/13/4.46, 48/hcp/12/4.95
145	$Pt_{116}O_{72}$	48/bridge/9/4.33, 24/fcc/13/5.23
146	$Pt_{116}O_{72}$	24/trough/13/4.46, 24/fcc/10/4.14, 24/fcc/13/5.23
147	$Pt_{116}O_{80}$	48/bridge/9/4.33, 24/fcc/10/4.14, 8/hcp/16/6.41
148	$Pt_{116}O_{80}$	24/trough/13/4.46, 48/hcp/12/4.95, 8/hcp/16/6.41
149	$Pt_{116}O_{84}$	12/bridge/8/3.89, 24/trough/13/4.46, 48/hcp/12/4.95
150	$Pt_{116}O_{84}$	48/bridge/9/4.33, 12/bridge/8/3.89, 24/fcc/13/5.23
151	$Pt_{116}O_{92}$	12/bridge/8/3.89, 24/trough/13/4.46, 48/hcp/12/4.95, 8/hcp/16/6.41
152	$Pt_{116}O_{96}$	48/bridge/9/4.33, 24/fcc/10/4.14, 24/fcc/13/5.23

Table S2: (continued)

#	Formula	Configuration (N_s/Type/CN/$\overline{\mathrm{CN}}$)
153	$Pt_{116}O_{96}$	48/bridge/9/4.33, 48/hcp/12/4.95
154	$Pt_{116}O_{104}$	48/bridge/9/4.33, 48/hcp/12/4.95, 8/hcp/16/6.41
155	$Pt_{116}O_{108}$	48/bridge/9/4.33, 12/bridge/8/3.89, 48/hcp/12/4.95
156	$Pt_{116}O_{116}$	48/bridge/9/4.33, 12/bridge/8/3.89, 48/hcp/12/4.95, 8/hcp/16/6.41
157	$Pt_{201}O_1$	1/bridge/9/4.39
158	$Pt_{201}O_1$	1/bridge/9/4.44
159	$Pt_{201}O_1$	1/fcc/11/4.59
160	$Pt_{201}O_1$	1/bridge/11/5.5
161	$Pt_{201}O_1$	1/trough/13/4.5
162	$Pt_{201}O_1$	1/hcp/12/5.09
163	$Pt_{201}O_1$	1/fcc/15/6.41
164	$Pt_{201}O_1$	1/hcp/14/5.95
165	$Pt_{201}O_1$	1/fcc/13/5.5
166	$Pt_{201}O_1$	1/hcp/16/6.95
167	$Pt_{201}O_1$	1/ontop/6/4.25
168	$Pt_{201}O_1$	1/ontop/7/5.0
169	$Pt_{201}O_1$	1/ontop/7/5.17

Table S2: (continued)

#	Formula	Configuration (N_s/Type/CN/$\overline{\mathrm{CN}}$)
170	$Pt_{201}O_1$	1/ontop/8/6.33
171	$Pt_{201}O_1$	1/ontop/9/7.5
172	$Pt_{201}O_{24}$	24/bridge/9/4.44
173	$Pt_{201}O_{24}$	24/hcp/14/5.95
174	$Pt_{201}O_{24}$	24/fcc/13/5.5
175	$Pt_{201}O_{48}$	24/fcc/11/4.59, 24/trough/13/4.5
176	$Pt_{201}O_{48}$	48/fcc/11/4.59
177	$Pt_{201}O_{48}$	24/bridge/9/4.44, 24/fcc/13/5.5
178	$Pt_{201}O_{48}$	24/bridge/9/4.44, 24/fcc/15/6.41
179	$Pt_{201}O_{48}$	48/bridge/9/4.39
180	$Pt_{201}O_{48}$	48/hcp/12/5.09
181	$Pt_{201}O_{48}$	24/bridge/9/4.44, 24/trough/13/4.5
182	$Pt_{201}O_{48}$	24/trough/13/4.5, 24/hcp/14/5.95
183	$Pt_{201}O_{72}$	48/fcc/11/4.59, 24/trough/13/4.5
184	$Pt_{201}O_{72}$	24/fcc/11/4.59, 24/trough/13/4.5, 24/hcp/12/5.09
185	$Pt_{201}O_{72}$	48/fcc/11/4.59, 24/fcc/15/6.41
186	$Pt_{201}O_{72}$	48/bridge/9/4.39, 24/fcc/15/6.41

Table S2: (continued)

#	Formula	Configuration (N_s/Type/CN/$\overline{CN}$)
187	$Pt_{201}O_{72}$	24/bridge/9/4.44, 24/trough/13/4.5, 24/fcc/15/6.41
188	$Pt_{201}O_{72}$	48/bridge/9/4.39, 24/hcp/14/5.95
189	$Pt_{201}O_{72}$	24/bridge/9/4.44, 48/hcp/12/5.09
190	$Pt_{201}O_{72}$	24/bridge/9/4.44, 24/trough/13/4.5, 24/fcc/13/5.5
191	$Pt_{201}O_{72}$	24/bridge/9/4.44, 24/trough/13/4.5, 24/hcp/16/6.95
192	$Pt_{201}O_{72}$	48/bridge/9/4.39, 24/bridge/9/4.44
193	$Pt_{201}O_{72}$	24/trough/13/4.5, 24/hcp/14/5.95, 24/fcc/13/5.5
194	$Pt_{201}O_{72}$	48/bridge/9/4.39, 24/bridge/11/5.5
195	$Pt_{201}O_{88}$	48/fcc/11/4.59, 24/trough/13/4.5, 8/fcc/15/6.41, 8/hcp/16/6.95
196	$Pt_{201}O_{96}$	48/bridge/9/4.39, 48/fcc/11/4.59
197	$Pt_{201}O_{96}$	24/bridge/9/4.44, 24/trough/13/4.5, 48/hcp/12/5.09
198	$Pt_{201}O_{96}$	48/bridge/9/4.39, 24/bridge/9/4.44, 24/fcc/13/5.5
199	$Pt_{201}O_{96}$	48/fcc/11/4.59, 24/trough/13/4.5, 24/fcc/15/6.41
200	$Pt_{201}O_{96}$	48/bridge/9/4.39, 24/bridge/9/4.44, 24/fcc/15/6.41
201	$Pt_{201}O_{96}$	48/bridge/9/4.39, 24/bridge/9/4.44, 24/hcp/14/5.95
202	$Pt_{201}O_{96}$	24/bridge/9/4.44, 24/trough/13/4.5, 24/hcp/14/5.95, 24/fcc/13/5.5
203	$Pt_{201}O_{96}$	48/bridge/9/4.39, 24/bridge/9/4.44, 24/bridge/11/5.5

Table S2: (continued)

#	Formula	Configuration (N_s/Type/CN/$\overline{\mathrm{CN}}$)
204	$Pt_{201}O_{120}$	48/bridge/9/4.39, 48/fcc/11/4.59, 24/fcc/15/6.41
205	$Pt_{201}O_{120}$	48/bridge/9/4.39, 48/fcc/11/4.59, 24/fcc/13/5.5
206	$Pt_{201}O_{120}$	48/fcc/11/4.59, 24/trough/13/4.5, 24/fcc/15/6.41, 24/fcc/13/5.5
207	$Pt_{201}O_{120}$	48/bridge/9/4.39, 48/fcc/11/4.59, 24/bridge/11/5.5

Table S3: Averaged Pt-Pt nearest-neighbor distances $\langle r_{\mathrm{Pt-Pt}} \rangle$ (in Å), averaged Pt-Pt coordination numbers $\langle \mathrm{CN}_{\mathrm{Pt-Pt}} \rangle$, averaged Pt-O nearest-neighbor distances $\langle r_{\mathrm{Pt-O}} \rangle$ and averaged Pt-O coordination numbers $\langle \mathrm{CN}_{\mathrm{Pt-O}} \rangle$ calculated for different model systems.

System	$\langle r_{\mathrm{Pt-Pt}} \rangle$	$\langle \mathrm{CN}_{\mathrm{Pt-Pt}} \rangle$	$\langle r_{\mathrm{Pt-O}} \rangle$	$\langle \mathrm{CN}_{\mathrm{Pt-O}} \rangle$
Pt_{38}	2.73	7.6	-	-
Pt_{79}	2.75	8.5	-	-
Pt_{116}	2.75	8.9	-	-
Pt_{201}	2.77	9.4	-	-
fcc Pt bulk	2.77	12.0	-	-
$Pt_{38}O_{76}$	3.13	2.2	2.01	4.8
$Pt_{79}O_{158}$	3.14	1.8	2.01	5.0
$Pt_{116}O_{232}$	3.11	1.8	2.01	5.1
$Pt_{201}O_{402}$	3.14	1.8	2.02	5.2
β-PtO_2 bulk	3.18	2.0	2.03	6.0

Table S4: Formation energies of the Pt nanoparticles and two extended Pt surfaces relative to bulk fcc Pt, and the formation energies PtO_2 nanoparticles relative to the formation energy of bulk PtO_2, each in eV per Pt atom.

System	Formation energy
Pt_{38}	1.13
Pt_{79}	0.84
Pt_{116}	0.75
Pt_{201}	0.58
fcc Pt(100)	0.58
fcc Pt(111)	0.40
$Pt_{38}O_{76}$	1.31
$Pt_{79}O_{158}$	1.04
$Pt_{116}O_{232}$	0.92
$Pt_{201}O_{402}$	0.81

Listing S1: Structure of the bulk β-PtO_2 (in CIF format) used to create the PtO_2 nanoparticles

```
# data source: https://doi.org/10.17188/1189224
data_PtO2
_symmetry_space_group_name_H-M   'P 1'
_cell_length_a   3.19178800
_cell_length_b   4.55466800
_cell_length_c   4.61138100
_cell_angle_alpha   90.00000000
_cell_angle_beta   90.00000000
_cell_angle_gamma   90.00000000
_symmetry_Int_Tables_number   1
_chemical_formula_structural   PtO2
_chemical_formula_sum   'Pt2 O4'
_cell_volume   67.03811115
_cell_formula_units_Z   2
loop_
 _symmetry_equiv_pos_site_id
 _symmetry_equiv_pos_as_xyz
  1  'x, y, z'
loop_
 _atom_site_type_symbol
 _atom_site_label
 _atom_site_symmetry_multiplicity
 _atom_site_fract_x
 _atom_site_fract_y
 _atom_site_fract_z
 _atom_site_occupancy
  Pt  Pt0  1  0.00000000  0.00000000  0.00000000  1
  Pt  Pt1  1  0.50000000  0.50000000  0.50000000  1
  O  O2  1  0.00000000  0.36239000  0.25916500  1
  O  O3  1  0.00000000  0.63761000  0.74083500  1
  O  O4  1  0.50000000  0.13761000  0.75916500  1
  O  O5  1  0.50000000  0.86239000  0.24083500  1
```
